# Transcriptome sequencing of rhizome tissue of *Sinopodophyllum hexandrum* at two temperatures

**DOI:** 10.1186/1471-2164-15-871

**Published:** 2014-10-07

**Authors:** Anita Kumari, Heikham Russiachand Singh, Ashwani Jha, Mohit Kumar Swarnkar, Ravi Shankar, Sanjay Kumar

**Affiliations:** Biotechnology Division, CSIR-Institute of Himalayan Bioresource Technology, PO Box No. 6, Palampur, 176 061 Himachal Pradesh India; Studio of Computational Biology & Bioinformatics, CSIR-Institute of Himalayan Bioresource Technology, PO Box No. 6, Palampur, 176 061 Himachal Pradesh India; Academy of Scientific & Innovative Research, New Delhi, India

**Keywords:** Below ground, Deep sequencing, Development, Gene expression, Growth, Next generation sequencing, Podophyllotoxin, RNA-seq, Stress

## Abstract

**Background:**

*Sinopodophyllum hexandrum* is an endangered medicinal herb, which is commonly present in elevations ranging between 2,400–4,500 m and is sensitive to temperature. Medicinal property of the species is attributed to the presence of podophyllotoxin in the rhizome tissue. The present work analyzed transcriptome of rhizome tissue of *S. hexandrum* exposed to 15°C and 25°C to understand the temperature mediated molecular responses including those associated with podophyllotoxin biosynthesis.

**Results:**

Deep sequencing of transcriptome with an average coverage of 88.34X yielded 60,089 assembled transcript sequences representing 20,387 unique genes having homology to known genes. Fragments per kilobase of exon per million fragments mapped (FPKM) based expression analysis revealed genes related to growth and development were over-expressed at 15°C, whereas genes involved in stress response were over-expressed at 25°C. There was a decreasing trend of podophyllotoxin accumulation at 25°C; data was well supported by the expression of corresponding genes of the pathway. FPKM data was validated by quantitative real-time polymerase chain reaction data using a total of thirty four genes and a positive correlation between the two platforms of gene expression was obtained. Also, detailed analyses yielded *cytochrome P450s*, *methyltransferases* and *glycosyltransferases* which could be the potential candidate hitherto unidentified genes of podophyllotoxin biosynthesis pathway.

**Conclusions:**

The present work revealed temperature responsive transcriptome of *S. hexandrum* on Illumina platform. Data suggested expression of genes for growth and development and podophyllotoxin biosynthesis at 15°C, and prevalence of those associated with stress response at 25°C.

**Electronic supplementary material:**

The online version of this article (doi:10.1186/1471-2164-15-871) contains supplementary material, which is available to authorized users.

## Background

*Sinopodophyllum hexandrum* (Royle) T.S. Ying, commonly known as Himalayan Mayapple, is an alpine herb of family Berberidaceae that grows on altitudes ranging between 2,400–4,500 m above mean sea level [1]. The species is a perennial, erect, and unbranched herb reaching to a height of 40–50 cm; rhizome gives rise to multiple stems. Rhizome is non-edible, but is the source of a cytotoxic compound podophyllotoxin which is an aryltetralin lignan. Cytotoxicity of the compound is reduced for safe clinical use through its transformation into several derivatives. Some of the derivatives such as etoposide (VP-16) and teniposide (VM-26) have anti-cancerous activities and hence podophyllotoxin is in much demand in pharmaceutical industry [2]. Extensive harvesting of the species from nature to meet the requirement of rhizome for podophyllotoxin extraction has led *S. hexandrum* to be listed as endangered species (Appendix II of convention for international trade in endangered species; http://www.cites.org/eng/app/appendices.php).

Several studies have reported on propagation and conservation of the species [3], podophyllotoxin biosynthesis [4], derivatization of podophyllotoxin, and their mode of action [5]. Also, a few studies have been carried out on plant response to environmental cues. Like other alpine plant species [6], *S. hexandrum* has been reported to be sensitive to high temperature [7]. *S. hexandrum* consumed more starch at 25°C as compared to at 10°C leading to poor root growth at the former temperature [7]. Also, the rate of photosynthesis was reported to be lower at 30°C as compared to at 20°C. Higher temperature was also reported to increase the transpiration rate and reduce water use efficiency indicating the species to be sensitive at high temperature [8]. We conducted a preliminary experiment to observe the growth of *S. hexandrum* at four different temperatures. *S. hexandrum* showed partial leaf folding and leaf drooping at 10°C and 35°C, respectively within a span of 3 weeks of temperature exposure, whereas the species did not exhibit such symptoms at 15°C and 25°C; though after 2–3 weeks of temperature, leaves lost shine and developed violet coloured spots at 25°C (Additional file 1). The species did not survive for long at 10°C and 35°C.

The objective of the present work was to study the molecular processes at 15°C and 25°C in commercially important organ rhizome to understand why the species performed better at 15°C. Transcriptome analysis offers a convenient route to understand molecular processes of the tissue in question. For example, analysis of populus (*Populus tremula*) transcriptome showed the importance of auxin and gibberellins signaling, and starch metabolism during winters [9]; in sessile oaks [*Quercus petraea* (Matt.) Liebl.], transcriptome analysis suggested the importance of cell rescue and defense during bud burst after dormancy [10]; subtracted transcriptome analysis in tea [*Camellia sinensis* (L.) O. Kuntze] identified the significance of genes involved in cell cycle/cell division and stress-inducible genes including those encoding chaperons during winter dormancy [11]. Next generation sequencing has eased transcriptome analysis, and the technology has been successively used to study the transcriptome of several plant species including Himalayan plant species *Picrorhiza kurrooa* ([12] and the references mentioned therein) to discover genes and markers vis-à-vis to gain insight to important biological and molecular processes. More so, temperature responsive transcriptome of *S. hexandrum* rhizome is not yet reported.

## Methods

### Plant material

Plants of *S. hexandrum* were collected from Parashar lake, Mandi (2,730 m above sea level; 30°12′ N 77°47′ E, India) and maintained at the institute in Palampur (1,300 m altitude; 32°06′ N, 76°33′ E, India) for one year in a polyhouse. During the collection procedure, the plants with rhizome of the similar size were selected for various experiments (India’s Biological Diversity Act 2002 permits access of biological resources to bonafide Indians for scientific research [13]). Plants from the polyhouse were shifted to two separate plant growth chambers (Percival Scientific, USA) maintained at 15°C and 25°C with 16 h photoperiod at a photosynthetic photon flux density of 200 μmol m^-2^ s^-1^. Plants were irrigated adequately and the rhizome tissue was harvested at day 0, 14, and 21 of transfer, cut quickly into smaller pieces using a sharp razor, mixed the cut pieces, frozen in liquid nitrogen and stored at -80°C for further analyses.

### Extraction and estimation of podophyllotoxin

Frozen samples were ground to fine powder in liquid nitrogen using pestle and mortar followed by addition of 700 μl of 70% methanol for 100 mg tissue with intermittent grinding [14–17]. Extract was transferred to a centrifuge tube and the pestle and mortar was rinsed with 300 μl of 70% methanol to recover the left over sample. Extracts were pooled, vortexed for 5 minutes, sonicated (Ultrasonic Cleaner, MC-109-MP, Oscar, India) for 10 minutes at 25°C and centrifuged at 14,000 rpm for 5 minutes to collect supernatant for podophyllotoxin estimation. Supernatant was filtered through 0.22 μm filter (Millipore, USA) to estimate podophyllotoxin on an Ultra Performance Liquid Chromatography (UPLC) system. The system consisted of Acquity UPLC (Waters, Millford, USA) equipped with binary solvent manager, sample manager, photodiode array detector and a bridged ethyl hybrid workflow shield C18 (1.7 μm particles, 2.1 × 100 mm) analytical column (Waters Corp., Manchester, UK). Mobile phase consisted of methanol and water in a ratio of 60:40. Isocratic elution was carried out at a flow rate of 0.250 ml min^-1^ with injection volume of 5 μl. Podophyllotoxin was monitored at 240 nm and quantified using standard podophyllotoxin (Sigma, USA). Three separate biological replicates were used for each estimation. Significant difference in podophyllotoxin content at 15°C and 25°C was calculated using Completely Randomized Design to obtain p-value at different sampling times.

### RNA extraction, cDNA preparation and transcriptome sequencing

Various protocols were followed essentially as described previously [12]. In brief, total RNA was extracted from rhizome tissue as described by Muoki et al. [18]. Nanodrop 1000 (NanoDrop Technologies, USA) and a Bioanalyzer Chip RNA 7500 series II (Agilent Technologies, USA) were used to determine quality and quantity of RNA. Oligotex mRNA Midi Kit (Qiagen, Germany) was used to purify mRNA from total RNA, and non-directional Illumina RNA sequencing library was prepared with mRNA-Seq 8 Sample Prep Kit (Illumina, USA) using random primers. Library had an average insert size of 200 bp and eight picomoles of the library [quantified using a Bioanalyzer Chip DNA 12000 series II (Agilent Technologies, USA)] was used for cluster generation. Paired end (PE) 36 × 2 bp sequencing was carried out on Illumina Genome Analyzer IIx (Illumina, USA) as per the manufacturer’s instructions.

### Expression analysis and validation with quantitative real-time reverse transcriptase mediated polymerase chain reaction (qRT-PCR)

Total RNA was isolated as described previously [18]. RNA was pretreated with RNase-free DNase I (Invitrogen, USA) and reverse transcribed with 1 μg of total RNA using Superscript III (Invitrogen, USA) according to the manufacturer’s instructions. Gene specific expression primers for qRT-PCR were designed using GenScript Real-time PCR Primer Design tool [19]. qRT-PCR was performed with three separate biological replicates on a Stratagene Mx3000P System (Agilent Technologies, USA) using 2× Brilliant II SYBR^®^ Green QPCR Master Mix (Agilent Technologies, USA). PCR was conducted under the following conditions: 10 min at 95°C (enzyme activation), 40 cycles each of 30s at 95°C, 30s at desired Tm (Additional file 2 has details), and 72°C for 30s. A final melting curve analysis was performed (55°C to 95°C) to verify specificity of amplicons. Transcript level of all the genes was normalized to an internal reference *actin*. Expression was estimated using Relative Expression Software Tool [20]. Expression values were transformed (log_2_) to generate expression profiles. All primers used in this study are listed in Additional file 2.

### *De novo*assembly and sequence clustering

**Figure 1:**
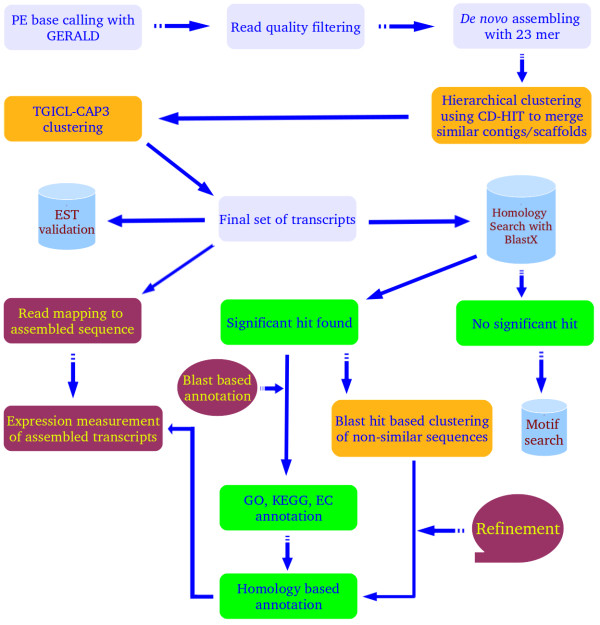
**Computational pipeline for analyzing transcriptome of** ***S. hexandrum.*** Methods section has details for various steps.

PE sequence reads were generated in FASTq format using CASAVA package GERALD tool provided by Illumina. Our previous publication by Gahlan et al. [12] described strategies and various protocols used for assembly and clustering. 36 × 2 bp PE reads were generated for individual sample in each lane. The last three bases from the 3′ end of each read were removed to minimize the sequencing error, which is usually higher in the 3′ end of reads. An in-house developed tool, filteR [12], was used to filter out the poor quality reads as well as adapter contaminated reads. *De novo* assembling of high quality reads was performed using SOAP*denovo* tool [21]. A k-mer size of 23 was opted for assembling which yielded high quality PE reads (Additional file 3). PE option of assembling with spacing distance of 200 bp between the PE read was applied to achieve more effective assembling using fragment library size information of PE reads. The same information was also used to build the scaffold sequences by merging two contigs into single scaffold sequence, sharing the read pairs. Figure 1 shows the protocol used in *de novo* assembling and transcript analysis of assembled sequences for a given sample. Sequence redundancy was removed by searching for similar sequences, keeping minimum similarity cut-off of 95% for CD-HIT-EST [22]. CD-HIT-EST was used for further clustering with 90% similarity cut-off. This clustering step was followed by overlap based assembling/clustering, using TGICL-CAP3. It was run with parameters like terminal overlap for at least 40 bp and 90% identity. The resulting singletons and consensus contigs were merged to get the final list of assembled transcripts. A *de-Bruijn* graph building approach was used for primary assembling in the initial stage of assembling, which provided long assembled sequences. These long assembled sequences were further taken for redundancy removal and assembling using approaches based on overlap layout consensus and compositional similarity based redundancy crunching. A set of scripts was developed to detect contigs/scaffolds having no sequence similarity but belonged to same gene’s different regions. Such sequences were clustered together to represent as a single transcript or unigene. The highest scoring BLASTX hit for all contigs were analyzed for common Non Redundant (NR) ID for a particular gene. All associated contigs showing highest similarity to the same gene but different locations were assigned a single common unigene identification. This approach of Dissimilar Sequence (DS) clustering minimizes the inflated reporting of total unique genes [12].

Information regarding the assembled transcript sequences, associated filtered read data and transcript grouping for unigenes, is available at http://scbb.ihbt.res.in/Podo-12-12-11/. Entire computational analysis was carried out on CentOS Linux based 48 cores 2.2 Ghz AMD processors server with 256 GB random access memory.

### Assembly validation

To validate assembled transcripts, BLASTN of 1,463 ESTs, downloaded from National Centre for Biotechnology Information (NCBI) dbEST, was performed against transcriptome of *S. hexandrum* using E-value cut-off of 1e^-05^. Also, selected unigenes involved in podophyllotoxin biosynthesis were validated after DS clustering against complete coding sequences of corresponding genes of *S. hexandrum* submitted in GenBank.

### Sequence annotation

Assembled transcripts of *S. hexandrum* were searched against UniProt database [23], Gene Ontology (GO) [24], Kyoto Encyclopedia of Genes and Genomes (KEGG) [25] and Enzyme Commission number (EC) [26], using BLASTX with a cut-off E-value of 10^-1^. Lower cut-off during annotation facilitates annotation even for instances that display only small region matching for some functional domain, which could be missed otherwise with higher cut-off values. Associated GO term was assigned for each assembled transcript by analyzing the GO term for the Uniprot sequence, which returned the highest scoring hit. Similar approach was followed for KEGG and EC classification and annotation for the assembled transcripts. Majority of GO, EC and KEGG based annotations and statistics were done using annotation tool, Annot8r [27] and an in-house developed scripts. Plant Transcription Factor Database (PlnTFDB) [28] hosts large number of plant specific transcription factors (TFs), their classification, corresponding nucleic acids and protein sequences. Data for all TFs reported in PlnTFDB, version 3.0, were downloaded for the current study. The assembled transcript sequences were searched against this database using BLASTX with an E-value threshold of 10^-5^. Top scoring homologous sequences from PlnTFDBs were used to annotate the assembled transcripts displaying highest similarity.

*S. hexandrum* transcript sequences were searched against MIPS Functional Catalogue (FunCat) [29] database to classify transcripts based on functional categories classified at MIPS. FunCat hierarchy is based on the *Arabidopsis thaliana* transcriptome. Therefore, BLASTX search was performed against the Arabidopsis transcriptome (downloaded from TAIR 10 [30]). The IDs from top scoring Arabidopsis gene hits were extracted and mapped to the *S. hexandrum* transcript sequences to categorize them into various FunCat categories.

### Functional domain search for unknown sequences

The assembled sequences which did not return any homologous sequence hit during BLASTX search were converted into six longest ORFs. These ORFs were scanned against the functional domain databases like Conserved Domain Database (CDD) [31], using RPS-BLAST UNIX version.

### Read mapping and transcript expression abundance measurement

Since a reference genome was not available in the present study, the filtered reads were mapped to the assembled transcripts. This was followed by estimation of total mapped reads. Uniquely mapped reads assigned to each assembled transcript with maximum two mismatches were allowed. Read mapping was done using TopHat which uses Bowtie to map the reads on transcripts [32] while differential abundance in terms of FPKM was measured using Cuffdiff [33] which deploys t-test and Benjamini-Hochberg correction to compute significance difference between samples and adjusts the p-value after false discovery rate for multiple hypothesis testing. Using the reads from 15°C and 25°C experiments, the relative abundance of transcripts was estimated for each unigene for the two temperatures for the sequence clusters generated following the DS clustering [12]. DS clustering reduced the inflation of unique genes representation and mapped that information on every part of the study. The read data obtained for the two temperatures were mapped on the assembled transcripts separately. Care was taken to ensure that only those lanes and reads were considered for both the temperatures which had equal sample concentration and produced almost equal percentage of reads qualifying the filtering cut-off, ensuring that experimental conditions do not skew the read representation and analysis.

The associated GO terms (GO terms are derived from dynamic controlled vocabularies or ontologies that can be used to describe the function of genes and gene products) and IDs were mapped to each of these unique transcripts and genes, providing single stop complete information regarding their related expressions at the two temperatures and their corresponding annotations.

### Guanine-cytosine (GC) content analysis and simple sequence repeats (SSRs) identification

GC content of the sequences was measured using Emboss GeeCee tool, while sequences were scanned for SSR markers using MISA [34].

### Metabolic networks and pathways analysis

Sequences were scanned against KEGG using BLAST. FPKM was considered for all the assembled sequences. Plant Metabolic Network (PMN) database [35] was searched for all the significantly up-regulated genes using an in-house developed network running script. Using the same script, pathways network images were generated and corresponding transcript, expression and other associated information were incorporated into the files.

## Results and discussion

Rhizome biology for *S. hexandrum* is of interest not only that rhizome is the source of medicinally important compound podophyllotoxin, but also it allows plant to survive the unfavorable environment of winters when aerial portion of the plant dies. Plant regenerates from the rhizome upon arrival of the growing season. Availability of vast genomic and transcriptomic data in the public domain coupled with next generation sequencing platforms made it possible to understand temperature responsive biology of rhizome of *S. hexandrum*. Since plants appeared healthier at 15°C as compared to at 25°C (Additional file 1), the present work analyzed transcriptome in the rhizome tissue at the two temperatures vis-à-vis podophyllotoxin content.

### Podophyllotoxin accumulation at two temperatures

**Figure 2:**
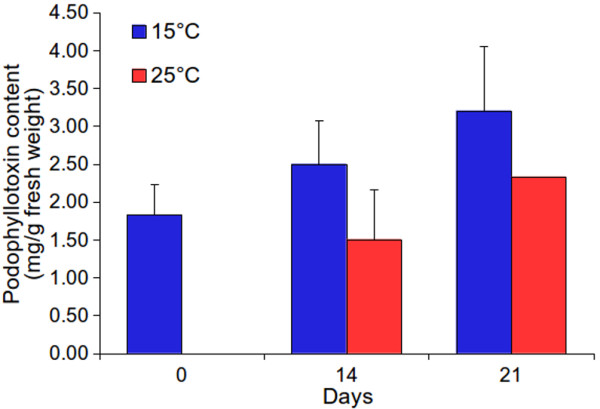
**Effect of temperature on accumulation of podophyllotoxin content in rhizome tissue of** ***S*** **.** ***hexandrum*** **.** Rhizome tissue was harvested at day 0, 14 and 21 from plants kept at 15°C and 25°C to measure podophyllotoxin content. Difference in the podophyllotoxin content at two temperatures was statistically insignificant as analyzed by Completely Randomized Design (CRD); though a decreasing trend of accumulation was observed at 25°C. Each value is a mean of three separate biological replicates. Error bars are standard error of the mean of three biological replicates.

Several studies showed modulation of secondary metabolism in response to external cues [36, 37]. In the present work podophyllotoxin content did exhibit a decreasing trend of accumulation, from day 14 and onwards of the exposure of plants to 25°C as compared to those at 15°C (p-values for day 14 and 21 were 0.393 and 0.686, respectively; F values for day 14 and 21 were 1.196 and 0.393, respectively) (Figure 2). Though there is no report on the effect of temperature on podophyllotoxin accumulation, one group [38] did report decreased accumulation of podophyllotoxin content at low (1,500 m) as compared to at high altitude (3,000 m) [38], where temperature is one of the major cues which is higher at lower altitude [39]. A decreasing trend of podophyllotoxin accumulation at higher temperature did suggest modulation of rhizome biology of *S. hexandrum* by temperature.

### Reads generation and *de novo*sequence assembly

A total of 146,304,154 PE reads were obtained at 15°C while a total of 54,176,602 PE reads were obtained at 25°C. By deploying a read filtering tool, FilteR [12], a total of 125,957,408 and 44,346,624 high quality PE reads were obtained for 15°C and 25°C, respectively (total 170,304,032 PE reads). Selection of appropriate k-mer size is an important step in *de novo* assembly, since it varies for species and data type. SOAP*denovo* was run to assemble transcripts of *S. hexandrum* from high quality reads at different k-mer size ranging from 19 to 29. K-mer size of 23 mer was chosen for *de novo* assembly as it displayed a balance between over- and under-represented transcript numbers, coverage of reads on transcripts, maximum length of transcript, percentage of transcripts having length higher than 1,000 bp and average transcript length (Additional file 3). Number of transcripts decreased linearly with increasing k-mer size suggesting under-representation at high k-mers and over-representation at lower k-mer length. Similarly, at high k-mer higher coverage or higher expression was observed for the assembled sequences. These steps were taken for evaluating the quality of assembled transcripts. Gap filling produced longer scaffolds from the PE reads data which mapped into the contigs as well as gapped regions. Collective assembly of the filtered reads yielded a total of 60,089 transcripts, 15.57% of which were above 1 kb, with a coverage of 88.34X, average length of 543.11 bp and maximum length of 14,390 bp (Table 1).

**Table 1:** **Summary of transcriptome data generated on Illumina Genome Analyzer IIx for rhizome tissue of** ***S*** **.** ***hexandrum***

	15°C	25°C	Total
Total number of paired end reads	146,304,154	54,176,602	200,480,756
Number of reads obtained after quality filtering	125,957,408	44,346,624	170,304,032
Number of primary assembled transcripts of pooled data	Not applicable	Not applicable	60,089
Average length of transcripts (bp) of pooled data	Not applicable	Not applicable	543.11
Average coverage (X) of pooled data	Not applicable	Not applicable	88.34

### Homology search, sequence clustering, validation of assembled transcripts and general traits of the transcriptome

The number of unique assembled transcripts reduced from 60,089 to 59,244 sequences after removing the redundancies. These were clustered together under a common Unigene group, and BLASTX [40] with cut-off E-value of 10^-5^ showed that a total 25,395 sequences had significant BLAST hit. Remaining 33,849 sequences without significant BLAST hit did show conserved domains in 761 sequences (Additional file 4), suggesting a possible function for these unknown sequences. DS clustering [12] further reduced the total sequences from 25,395 to 20,387 (Additional file 5; URL: http://scbb.ihbt.res.in/Podo-12-12-11/).

A total of 1,463 ESTs of *S. hexandrum* were reported in NCBI dbEST, out of which BLAST hits were found for 975 ESTs (66.64%) when searched against the transcriptome. These ESTs showed an average identity of ~93.50% indicating good quality of assembled transcripts. Of the total, 19.69% of ESTs (192/975) had coverage greater than 90% whereas 52.82% ESTs (515/975) showed coverage of 50% or more. To evaluate correct assembly of unigenes, coding sequences of five selected genes (accession numbers GU324975.1, EU240218.2, GU196273.1, KF170372.1 and GU228507.1) involved in podophyllotoxin biosynthesis pathway were searched against unigenes using BLASTN. Analysis yielded average coverage of 95.05% (Additional file 6), suggesting that the unigenes were correctly assembled.

Average GC content of *S. hexandrum* transcripts was found to be around 44.59% out of which 80% of transcripts had the content in the range of 40-49%, a range comparable to that reported for dicots (44-47%) [41] (Additional file 7). GC content of genome indicates several features including genome stability and possible repeats dynamics [42]. Assembled transcript sequences of *S. hexandrum* identified a total of 3,226 SSRs. Most abundant SSR group was of tri-nucleotide (54.40%) with GAA, TTC, TCT, CAC and CCA as predominant repeats, followed by di-nucleotide (36.38%) with prevalent occurrence of poly-CT (261), and mono-nucleotide (19.36%) SSRs represented by Poly-T (272) as a dominant repeat. Only a small fraction of tetra (32) and hexa (3) SSRs contributed to the pool (Additional file 7). SSRs are used in production and control of strains as well as dissemination of important genes apart from being the source of polymorphism and marker of genome [43].

### Functional annotation and classification of the *S. hexandrum*transcriptome

Annotation of 20,387 assembled sequences against GO database yielded significant annotation for 15,810 unique transcripts that were classified into biological processes, molecular functions and cellular component using plant specific GO slims. Functional classification in biological process category (Additional file 8) revealed that metabolic process, response to stimulus, cellular process and multicellular organismal development were among the highly represented groups, suggesting tissue to be metabolically active. Genes involved in DNA binding, catalytic and transferase activity were highly represented in molecular function category (Additional file 8), indicating dominance of gene regulation, signal transduction and enzymatically active processes. Among the cellular components (Additional file 8), genes for intracellular location and those encoding for membrane localized protein were most represented, which is a typical character of a eukaryotic cell.

Best EC classification was obtained for 8,172 assembled sequences after DS clustering. An analysis of top 50 abundant enzyme classes showed predominance of serine/threonine protein kinase enzyme (21.73%; Additional file 9A), which is known to be involved in regulation of cell proliferation, programmed cell death (apoptosis) and cell differentiation [44]. Associated KEGG classification could be obtained for 8,759 assembled sequences. An analysis of top 50 KEGG pathways showed that plant hormone signal transduction pathways (5.74%) dominated the group (Additional file 9B), followed by plant-pathogen interaction. GO, EC and KEGG analysis showed rhizome to be a metabolically active tissue.

### Global analysis of transcript abundance in response to temperature

**Figure 3:**
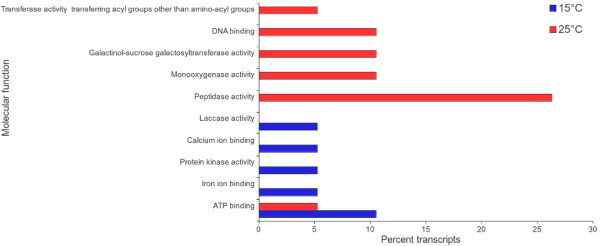
**Percent transcripts in transcriptome characterized for molecular function category which exhibit significant over-expression at 15°C and 25°C as compared to those at 25°C and 15°C, respectively.**

**Figure 4:**
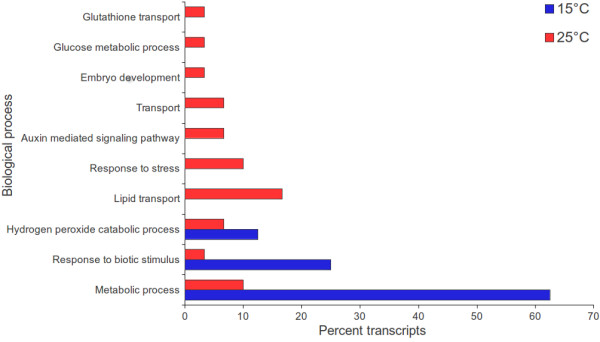
**Percent transcripts in transcriptome characterized for biological process category which exhibit significant over-expression at 15°C and 25°C as compared to those at 25°C and 15°C, respectively.**

Read mapping-based method of estimation of transcript abundance offers high throughput gene expression data, especially for those organism whose genome/transcriptome is not sequenced [12, 45]. GO annotation was obtained for 15,708 unique genes along with their FPKM values at the two different temperatures. Distribution of *S. hexandrum* unigenes across various molecular function categories (Figure 3) revealed prominent up-regulation of several genes including for protein kinase activity, and calcium ion binding at 15°C. While genes associated with monoxygenase activity, peptidase activity, galactinol-sucrose galactosyltransferase activity were over-expressed at 25°C. Of the various biological process categories (Figure 4), hydrogen peroxide catabolic process, and response to biotic stimulus were prominent at 15°C, whereas response to stress, and auxin mediated signaling pathway exhibited significant over expression at 25°C.

**Figure 5:**
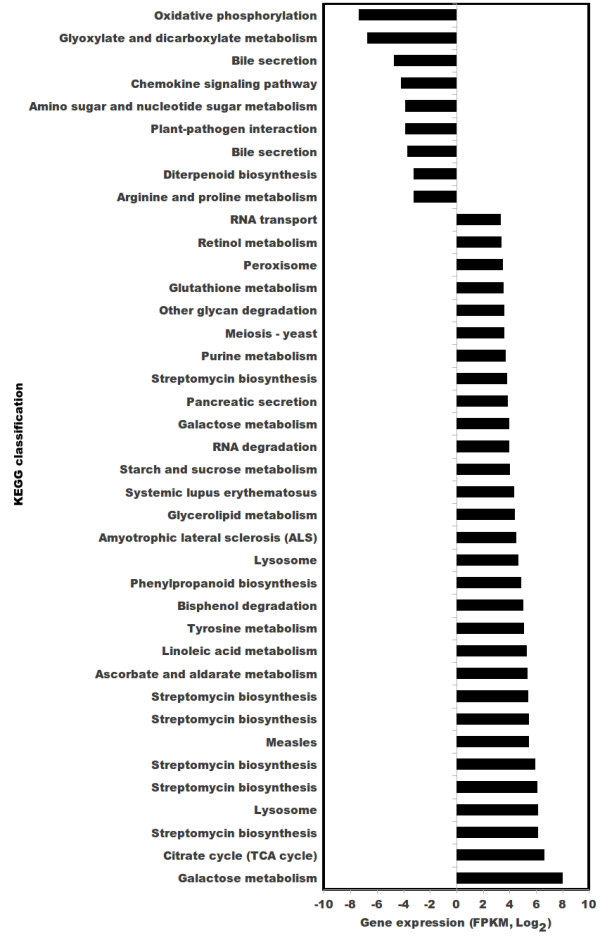
**Top 50 Kyoto Encyclopedia of Genes and Genomes (KEGG) pathways of significantly over- and under-expressed transcripts at 15°C as compared to at 25°C.**

KEGG identified pathways for 8,711 representative sequences, which were mapped for abundance and analyzed for their behavior at the two temperatures. Some of the pathways that exhibited significant over-expression at 15°C included those associated with citrate cycle and metabolism of ascorbate, sucrose and glutathione (Figure 5). Glutathione has been shown to have implications in heat, drought and cold responses [46]. An interesting feature was over-expression of phenylpropanoid (PP) biosynthesis pathway at 15°C, the pathway that supplies precursor for podophyllotoxin biosynthesis.

**Figure 6:**
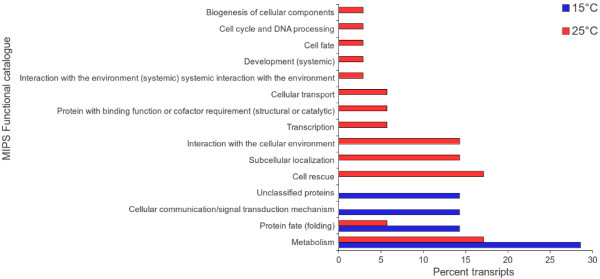
**Number of transcripts under various Functional Catalogue (FunCat) categories.** FunCat analysis was performed for significantly over-expressed transcripts at 15°C and 25°C as compared to those at 25°C and 15°C, respectively.

FunCat analysis (Figure 6; Additional file 10) revealed up-regulation at 25°C of genes involved in cell rescue, cell fate, cell cycle and DNA processing, apart from other genes. GO and FunCat analysis revealed that the genes associated with growth and development were over-expressed at 15°C. Whereas, genes involved in stress response, cell rescue and cell fate were expressed at 25°C.

PMN analysis using homologous genes exhibiting 2-fold or higher expression at 15°C is mentioned at http://scbb.ihbt.res.in/Podo-12-12-11/podo_pathway_go/Genes_at_15_degree_having_expression_2_fold_or_above/. Every network diagram contains the pathway information, associated homologous gene, transcript ID, and expression/abundance in terms of FPKM; each file name contains the associated transcript’s scaffold/contig ID in its prefix to make the browsing job easier. The file “podophyllum_analysis.doc” in the parent directory of the above link contains tabular representation and details about all such assembled transcripts, associated homologue reported in PMN, associated gene and associated PMN metabolic pathway/network.

Data showed up-regulation of pathways for respiration, photosynthesis, and phenylpropanoid biosynthesis at 15°C. These are fundamental pathways that would be crucial for growth and survival of plants at 15°C. Data showed up-regulation of ABA biosynthesis, ethylene and jasmonic acid, and is suggestive of their roles in plant growth and development at 15°C through functioning as signal molecule [47]. Up-regulation of phenylalanine metabolism, fatty acid biosynthesis, starch and sucrose metabolism and PP pathway at low temperature is also reported in cassava (*Manihot esculenta*) transcriptome [48]. This global analysis of gene expression provided a comprehensive data-set with each gene represented by its absolute expression level at two temperatures.

### Validation of FPKM-based analysis by quantitative real-time polymerase chain reaction (qRT-PCR)

FPKM based expression values were validated by qRT-PCR using sixteen genes related to growth and development and stress response. These genes were *NEDD8*, *CTPS*, *ATHB-14*, *ABTB2*, *AP2*, *CO*, *CSLA*, *CPN60*, *ZFP*, *AOX1a*, *STK*, *HSP60*, *HSP*, *AP2D*, *FRO* and *TINY*. Cullin-associated NEDD8-dissociated protein 1 regulates cell division, stress responses and hormonal signaling [49]. *CTP synthase* is involved in embryo development [50]. Homeobox-leucine zipper protein ATHB-14 (HDZip) family of TFs acts in developmental processes and in the mediation of external signals to regulate plant growth [51]. *ABTB*2, *AP2* and *CO* were reported to play a role in morphogenesis in Arabidopsis [52–54]. *CSLA* is required for synthesis of cell wall polysaccharide mannan which serves as storage reserve during plant growth and development [55]. *CPN60* was essential for development of embryo and seedling [56].

**Figure 7:**
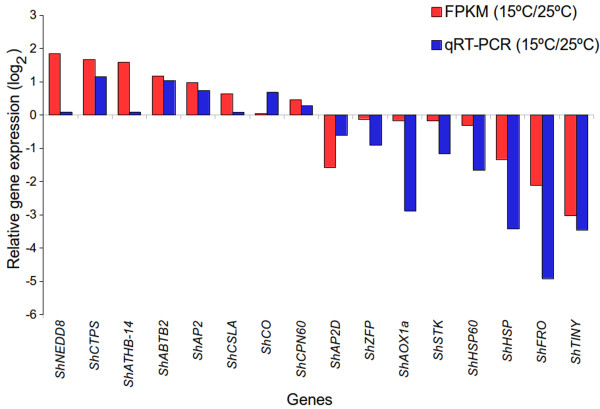
**Relative expression of sixteen genes associated with growth and development and stress response at 15°C as compared to those at 25°C based upon the data obtained by fragments per kilobase of exon per million fragments mapped (FPKM) values and validated by quantitative reverse transcriptase-polymerase chain reaction (qRT-PCR).** Name of each gene starts with a prefix *Sh* that stands for *Sinopodophyllum hexandrum*. Correlation coefficient of FPKM and qRT-PCR was 0.811 (p-value = 7.86 e^-05^). Full name of genes, FPKM data, primers and qRT-PCR condition are given in Additional file 2, Sheet 1.

*ZFP* plays an important role in stress signaling in Arabidopsis [57]. *AOX1a* was reported to prevent formation of excessive reactive oxygen species under stress condition [58]. *STK* is expressed in response to biotic and abiotic stress [59]. *HSP60* and *HSP*s encode proteins that prevent damage to cellular protein in response to heat stress [60]. *AP2D* is involved in abiotic stress [61]. *FRO* is required for iron transport across membranes for efficient photosynthesis [62]. *TINY* is associated with both abiotic and biotic stress signalling pathway and its over-expression suppressed cell proliferation [63]. FPKM and qRT-PCR based expression data were in accordance with each other with a correlation coefficient of 0.811 (p-value = 7.86 e^-05^) (Figure 7; Additional file 2). These values are considered significant [64, 65] and offer confidence to use FPKM based expression values to represent change in gene expression.

### Transcription factor and expression enrichment

TFs are sequence specific DNA-binding proteins that interact with the promoter regions of target genes and modulate gene expression. These proteins regulate gene transcription depending upon tissue type and in response to internal signals, for example, plant hormones, and to external signals such as temperature, UV light, pathogen attack and drought. Coordinated transcriptional control of genes is one of the major mechanisms regulating various processes [12]. In *S. hexandrum* 16,473 transcript sequences exhibited homology with TF families, which was reduced to 7,853 after DS clustering. The most abundant TF families observed were those encoding for C3H, bHLH, PHD, C2H2, AP2-EREBP and MYB (Additional file 11). Of the 7,853 *TFs*, 0.44% were found to be significantly over expressed at 15°C (Additional file 12). The most abundant *TFs* in this group were *WRKY*, *HB*, *SET*, *Orphans*, *MYB*, *GRAS* and *bHLH*. A total of 0.41% *TFs* exhibited significant abundance increment at 25°C, and these were *C3H*, *C2H2*, *bZIP* and *AP2-EREBP*. These *TFs* have been reported to play a role in stress response. The median fold expression was found higher for several TF families at 15°C (Table 2). TF bHLH is regulatory component involved in many developmental pathways as well as in plants metabolism [66]. MYB is implicated in response to development and metabolism [67]. TF PHD is a transcriptional regulator [68], and NAC regulates cell division and senescence [69]. C2H2 is associated with RNA metabolism and chromatin remodeling [70]. *TF* data showed operation of various cascades associated with growth and development at low temperature, while stress-responsive *TFs* were operative at high temperature (Table 2).

**Table 2:** **Transcription factor families exhibiting significant difference in expression (based upon FPKM values) at 15°C and 25°C in rhizome of** ***S*** **.** ***hexandrum***

***TF***Name	Median fold enrichment [15°C/25°C]	Median fold enrichment [25°C/15°C]
*ABI3VP1*	11.8496004672	0.084391031
*AP2-EREBP*	0.0993436445	10.0700936999
*ARF*	251.2693584621	0.0039797929
*BBR/BPC*	0.0222768823	44.8895849311
*bHLH*	34.9929358021	0.0285771964
*bZIP*	0.0168311473	59.6713654895
*C2H2*	0.0608432609	19.5386817714
*C3H*	0.0503718206	19.8523695815
*CCAAT*	0.0840696948	11.8948927191
*CSD*	0.0625543682	15.9860938481
*FAR1*	0.0644243725	15.5220759063
*FHA*	0.0438438485	22.8082167472
*G2-like*	83.8294374002	0.0119289838
*GRAS*	56.8761946615	0.0178378292
*HB*	24.9465837862	0.0409371239
*HMG*	271.7954207871	0.0036792379
*Jumonji*	6.002434472	13.3464369086
*LOB*	33.1564538771	0.0301600407
*MADS*	13.3186291414	0.0750828024
*MYB*	18.2000355303	0.0549449477
*MYB-related*	17.0809876818	0.0585446239
*NAC*	0.1193331901	8.3798983242
*Orphans*	45.9958631506	8.7044704786
*PHD*	0.0087271093	114.6268134388
*SET*	41.296776066	0.0341751871
*SNF2*	8.4420467469	26.9126761966
*SRS*	12.9995925944	0.0769254877
*Trihelix*	27.1957822459	0.0367704077
*VARL*	250.1042595646	0.0039983325
*WRKY*	17.1515127277	0.0583038952

Thus, transcriptome analysis including TF, GO, FunCat, EC and PMN data (Figures 3, 4, 5 and 6) suggested that 15°C favored growth and development, whereas 25°C imposed stress on *S. hexandrum*; and also supported the observation of better growth of plant at 15°C (Additional file 1).

### Expression of genes involved in podophyllotoxin biosynthesis at the two temperatures

**Figure 8:**
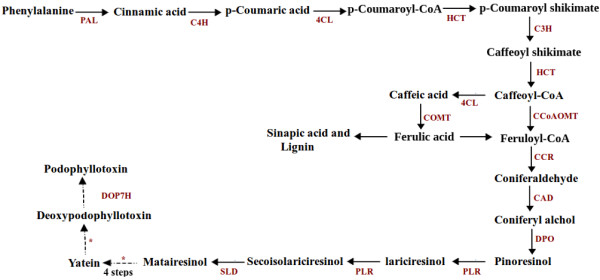
**Putative podophyllotoxin biosynthetic pathway in** ***S. hexandrum*** **(adapted from**[85, 91]**).** Solid arrow indicates known step whereas broken arrow indicates putative reaction; *shows uncharacterised step. Enzyme abbreviations are as follows: PAL, phenylalanine ammonia lyase; C4H, cinnamate 4-hydroxylase; 4CL, 4-coumarate: CoA ligase; HCT, *p*-hydroxycinnamoyl-CoA: quinate shikimate p-hydroxycinnamoyl transferase; C3H, *p*-coumarate 3-hydroxylase; CCoAOMT, caffeoyl-CoA O-methyltransferase; COMT: caffeic acid 3-O-methyltransferase; CCR, cinnamoyl-CoA reductase; CAD, cinnamyl alcohol dehydrogenase; DPO, dirigent protein oxidase; PLR, pinoresinol–lariciresinol reductase; SLD, secoisolariciresinol dehydrogenase; DOP7H, deoxypodophyllotoxin 7-hydroxylase.

Transcriptome data was used to identify various genes involved in the biosynthesis of podophyllotoxin, a lignan moiety. Glucose-6-phosphate is the central precursor for synthesis of a range of compounds in a living cell. The molecule produces phosphoenolpyruvate through glycolysis and erythrose-4-phosphate by oxidative pentose phosphate pathway. These two molecules synthesize chorismate through shikimate pathway. Chorismate synthesizes three aromatic amino acids tryptophan, phenylalanine and tyrosine out of which phenylalanine enters the PP pathway to synthesize *p*-coumaroyl-CoA (Figure 8). Bioconversion studies of radioactive precursors suggested coniferyl alcohol, a monolignol, to be the key precursor in the biosynthesis of podophyllotoxin [71]. Synthesis of coniferyl alcohol is achieved through several reactions starting from *p*-coumaroyl-CoA. One of the first enzymes in the sequence is *p*-hydroxycinnamoyl-CoA: quinate shikimate p-hydroxycinnamoyl transferase (HCT), which is known to catalyze two different steps. HCT catalyzes transfer of the *p*-coumaroyl group to shikimate [72] leading to formation of *p*-coumaroyl shikimate which in turn is hydroxylated by *p-*coumarate 3-hydroxylase (C3H) to produce caffeoyl shikimate [73]. Following the formation of caffeoyl shikimate, HCT also catalyzes transfer of caffeoyl moiety back to Coenzyme A yielding caffeoyl-CoA. Caffeoyl-CoA O-methyltransferase (CCoAOMT) methylates caffeoyl-CoA and synthesizes feruloyl-CoA followed by its reduction to synthesize coniferaldehyde by involving the action of cinnamoyl-CoA reductase (CCR). Last step in biosynthesis of lignin monomer is catalyzed by cinnamyl alcohol dehydrogenase (CAD), which catalyzes NADPH-dependent reduction of coniferaldehyde to coniferyl alcohol [74].

Studies on biosynthetic pathway of podophyllotoxin in *Podophyllum peltatum* and *Linum flavum* established occurrence of dirigent protein mediated coupling of coniferyl alcohol to get pinoresinol by involving the action of dirigent protein oxidase (DPO) [75]. Sequential conversion of pinoresinol to lariciresinol and secoisolariciresinol by the action of pinoresinol–lariciresinol reductase (PLR) was revealed by radiolabeled substrate study [76, 77]. Secoisolariciresinol is dehydrogenated to matairesinol by action of secoisolariciresinol dehydrogenase (SLD) [78]. The enzymatic reactions between matairesinol to deoxypodophyllotoxin are not well characterized. A direct pathway from matairesinol to yatein is proposed by tracer experiments in *Anthriscus sylvestris* [79] and pathway from yatein to podophyllotoxin via deoxypodophyllotoxin was suggested by feeding experiment [80], but not yet deciphered. Deoxypodophyllotoxin is considered as the precursor for biosynthesis of podophyllotoxin [81] as supported by feeding experiment in *S. hexandrum* [82] and *Linum flavum* [83, 84]. Hydroxylation of deoxypodophyllotoxin by deoxypodophyllotoxin 7-hydroxylase (DOP7H) involved in the biosynthesis of podophyllotoxin is yet to be characterized [85] (Figure 8).

**Figure 9:**
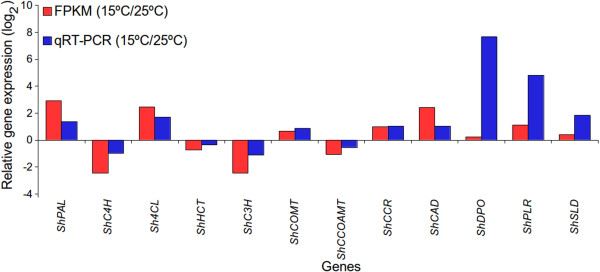
**Relative expression of genes associated with podophyllotoxin biosynthesis at 15°C relative to at 25°C.** Changes in the abundance of transcripts were analyzed by fragments per kilobase of exon per million fragments mapped (FPKM) as well as quantitative reverse transcriptase-polymerase chain reaction (qRT-PCR). Name of each gene starts with a prefix *Sh* that stands for *Sinopodophyllum hexandrum*. Correlation coefficient of FPKM and qRT-PCR was 0.740 (p-value = 0.009). *Actin* was used as an internal control and each value represents average of three separate biological replicates. Full name of genes, FPKM data, primers and qRT-PCR condition are given in Additional file 2, Sheet 2.

Starting from phenylalanine, there are twelve known genes involved in the biosynthesis of podophyllotoxin (Figure 8). Transcriptome data identified all these twelve genes. FPKM showed up-regulation of eight genes namely, *phenylalanine ammonia lyase* (*ShPAL*)*, 4-coumarate: CoA ligase* (*Sh4CL*), *caffeic acid 3-O-methyltransferase* (*ShCOMT*), *ShCCR*, *ShPLR*, *ShSLD*, *ShCAD and dirigent protein oxidase* (*ShDPO*) at 15°C as compared to at 25°C. Whereas, expression of *ShC4H*, *ShHCT*, *ShC3H* and *Sh*CCoAOMT showed down-regulation at 15°C. Validation of gene expression by qRT-PCR showed similar trend of gene expression as obtained by FPKM, with correlation coefficient of 0.740 (p-value = 0.009) between the two platforms for gene expression (Figure 9). Interestingly, the trend of podophyllotoxin content accumulation was higher at 15°C as compared to those at 25°C (Figure 2). Thus the results were in accordance with previous reports where gene expression and the metabolite concentration were positively correlated with each other. For example, catechins content in *Camellia sinensis* was positively correlated with the expression of genes of its biosynthetic pathway [86] and similar were the conclusions for picrosides content in *P. kurrooa* [87], steviosides content in *Stevia rebaudiana* [88] and shikonins content in *Arnebia euchroma* [89].

### Transcriptome data identified *cytochrome P450*(*CYP*s), *methyltransferases*(*MT*s) and *UDP*- *glycosyltransferases*(*UGT*s), possibly associated with the biosynthesis of podophyllotoxin

CYPs are membrane bound heme proteins, which catalyze hydroxylation, epioxidation, dealkylation and oxidation reactions. *CYPs* constitute the third known largest family of plant genes involved in numerous biosynthetic reactions resulting in production of plant hormones, defensive compounds, and secondary metabolites [90]. In podophyllotoxin biosynthetic pathway, hydroxylation at position 7 of deoxypodophyllotoxin by deoxypodophyllotoxin 7-hydroxylase (DOP7H) leads to the synthesis of podophyllotoxin, but the enzyme and the gene are yet to be characterized [82, 91]. Therefore, detailed analysis of *CYP*s was important.

Transcriptome data identified 116 *CYPs* based on highest bit score and significant E-value. *CYP-39* (scaffold11394_153.2) was found to be significantly up-regulated at 25°C by cuffdiff (Additional file 13). Fifteen *CYPs* showed more than two fold up-regulation, while 24 showed down-regulation at 15°C as compared to at 25°C (Additional file 14). These 39 *CYPs* could be the potential candidates for detailed analysis to identify the putative *ShDOP7H*.

MTs are transferase enzymes that participate in transfer of a methyl group from a donor to an acceptor. O-methylation plays important role in biosynthesis of lignan including podophyllotoxin [92]. Ferulic acid and sinapic acid are methylated compounds and are precursor of monolignols (coniferyl and sinapyl alcohols), the moieties involved in lignin biosynthesis [93]. MTs are essential in PP pathway: activity of CCoAOMT is essential for coniferyl and sinapyl alcohols biosynthesis [94]; and COMT is the key enzyme involved in methylation using hydroxycinnamates as substrates [95]. Tracer studies in *Anthriscus sylvestris* revealed that conversion of matairesinol to yatein involves four steps that include hydroxylation, dual methylation and methylenedioxy bridge formation [79]. Of the total 63 *MT*s identified, 18 *MT*s exhibited differential modulation by two fold and above (Additional file 13) wherein 2 *MTs* showed up-regulation, and 16 showed down-regulation at 15°C as compared to at 25°C (Additional file 15). Deeper analysis of these *MTs* could be promising to identify the genes associated with podophyllotoxin biosynthesis.

Glycosyltransferases (GTs) are super family of enzymes that catalyze transfer of sugars to a wide range of acceptor molecules. Glycosylation of natural products is generally catalyzed by UGTs of family 1 GTs [96]. In higher plants, UGT superfamily glycosylates a broad array of aglycones including plant hormones, plant secondary metabolites and xenobiotics such as herbicides [97]. Glycosylation contributes to increased water solubility, chemical stability and reduced chemical activity and thus plays a role in cell homeostasis, plant growth, development and in response to abiotic stress. Hydroxylated molecules are the common acceptors, while UDP-glucose is the most common donor in the GT catalyzed glycosyl group transferring reaction.

In the lignin biosynthesis pathway, lignin monomers like coumaryl, coniferyl alcohol and sinapyl alcohol are translocated in the form of glucosides from cytosol to cell wall. Involvement of UGTs in the biosynthesis of lignin was also reported [98]. Enzyme activity is expected to be essential for vacuolar storage of otherwise toxic lignans and is shown to be correlated with lignan glucoside accumulation. A podophyllotoxin-glucose glucosidase has been isolated from *Podophyllum peltatum* [99]. Thus it would be relevant to identify the UGTs involved in enzymatic synthesis of valuable glycoconjugates. BLAST search identified 35 unigenes encoding GTs. A total of 10 *UGT*s (Additional file 16) exhibited modulation by two fold and above at the two temperatures as analysed through FPKM (Additional file 13), of which 8 *UGT*s were down-regulated while 2 *UGT*s showed up-regulation at 15°C as compared to 25°C. In depth analysis of these modulated *UGT*s would be needed to identify the possible candidates associated with podophyllotoxin biosynthesis.

**Figure 10:**
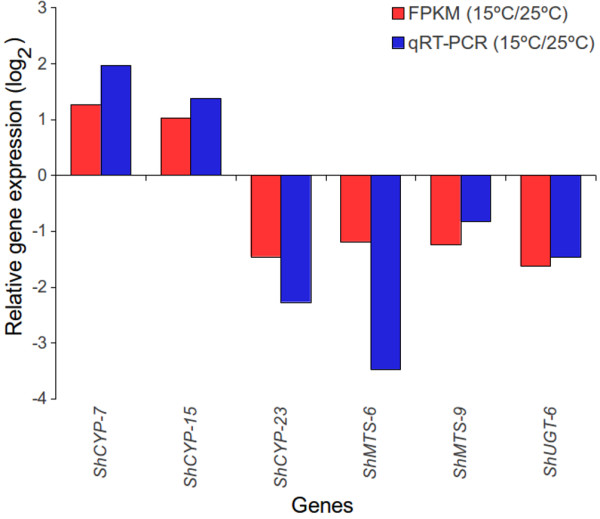
**Relative expression of selected** ***cytochrome P450*** **(** ***CYPs*** **), m** ***ethyltransferases*** **(** ***MTs*** **) and** ***UDP*** **-** ***glycosyltransferase*** **(** ***UGT*** **) at 15°C as compared to at 25°C based upon the data obtained by fragments per kilobase of exon per million fragments mapped (FPKM) values and validated by quantitative reverse transcriptase-polymerase chain reaction (qRT-PCR).** Name of each gene starts with a prefix *Sh* that stands for *Sinopodophyllum hexandrum*. Correlation coefficient of FPKM and qRT-PCR was 0.884 (p-value = 0.0194). *Actin* was used as an internal control and each value represents average of three separate biological replicates. Name of genes, FPKM data, primers and qRT-PCR condition are given in Additional file 2, Sheet 3.

FPKM based expression values of the selected genes were also validated by qRT-PCR. These genes were *ShCYP-7*, *ShCYP-15*, *ShCYP-23*, *ShMTS-6*, *ShMTS-9* and *ShUGT-6* (Additional file 2). *ShCYP-7*, and *ShCYP-15* were up-regulated, whereas *ShCYP-23*, *ShMTS-6*, *ShMTS-9*; and *ShUGT-6* were down-regulated at 15°C over 25°C. FPKM and qRT-PCR based expression data was in accordance with each other (Figure 10) with correlation coefficient of 0.884 (p-value = 0.0194) suggesting a significant agreement between the expression patterns observed through the two different platforms [64, 65].

## Conclusion

The present work analysed temperature (15°C and 25°C) responsive transcriptome of rhizomatous tissue of medicinally important endangered species *S. hexandrum*. A total of 60,089 assembled sequences, representing 20,387 unique genes were obtained on Illumina platform. Assembly of unigenes was validated by full-length sequence data of the available genes of *S. hexandrum,* which showed correct assembly. Transcriptome had an average coverage of 88.34X, average length of 543.11 bp, average GC content of 44.59% and abundance of trinucleotide SSR (54.40%).

Functional annotation and classification of the *S. hexandrum* transcriptome revealed metabolic process, response to stimulus, cellular process and multicellular organismal development to be the highly represented groups, suggesting rhizome to be metabolically active tissue.

FPKM data was validated by qRT-PCR data and the two platforms of gene expression showed significantly positive correlation emphasizing the confidence in the two different methods of gene expression. Data showed over-expression of genes and TFs associated with growth and development at 15°C, whereas those associated with stress tolerance over-expressed at 25°C. Also, various genes involved in podophyllotoxin biosynthesis were identified and their expression both by FPKM and the qRT-PCR showed up-regulation of eight out of twelve genes at 15°C. Interestingly, podophyllotoxin accumulation also showed increased trend of accumulation at 15°C. In-depth analyses of *CYPs*, *MTs* and *UGTs* yielded the potential candidate hitherto unidentified genes of podophyllotoxin biosynthesis. Data generated in the present work will contribute to future studies in the field of functional genomics and metabolic engineering for this important plant species. It will also be worthwhile to analyse microRNAs (miRNAs) because of their role in regulating gene expression either by blocking the process of translation or breaking the target transcripts [100]. MiRNAs are core regulatory component defining spatio-temporal states and are involved in stress responses as well. Challenge would be to analyse miRNAs in this species since its genome sequence is not yet reported [101].

## Availability of supporting data

Raw read files used for assembly were submitted to Short Read Archive (SRA) at NCBI under the accession ID SRX682844 (http://www.ncbi.nlm.nih.gov/sra/?term=SRX682844). Transcriptome sequences were submitted to Transcriptome Shotgun Assembly (TSA) project and deposited at DDBJ/EMBL/GenBank under the accession ID GBJY00000000 [http://www.ncbi.nlm.nih.gov/nuccore/GBJY00000000. The version described in this paper is the first version, GBJY01000000 (http://www.ncbi.nlm.nih.gov/Traces/wgs/?val=GBJY01)]. Sequences, which were less than 200 bp long or had a long stretch of “N” (>14 nucleotides) were not accepted by TSA. These sequences along with submitted sequences can be accessed at http://scbb.ihbt.res.in/Podo-12-12-11/.

## Electronic supplementary material

Additional file 1: **Leaf morphology of** ***S. hexandrum*** **grown at 10°C, 15°C, 25°C and 35°C for 21 days.** (PNG 1 MB)

Additional file 2: **Details on fragments per kilobase of exon per million fragments mapped (FPKM) values, and oligonucleotide sequences/primers, reaction conditions used in quantitative real-time polymerase chain reaction (qRT-PCR) for genes involved in growth, development and stress response (Sheet 1), podophyllotxin biosynthesis (Sheet 2), and selected** ***cytochrome P450*** **(** ***CYPs*** **),** ***methyltransferases*** **(** ***MTs*** **) and** ***UDP*** **-** ***glycosyltransferase*** **(** ***UGT*** **) (Sheet 3).** (XLS 36 KB)

Additional file 3: **Effect of k-mer size on assembling performance of transcriptome.** (DOC 31 KB)

Additional file 4: **Top 15 conserved domains identified by RPS-BLAST in** ***S. hexandrum*** **transcriptome.** (TIFF 2 MB)

Additional file 5: **List of scaffolds belonging to the different groups after dissimilar clustering step.** (XLS 2 MB)

Additional file 6: **Blast hit of unigenes involved in podophyllotoxin biosynthesis pathway against already submitted coding sequences in GenBank.** (XLS 16 KB)

Additional file 7: **Guanine-cytosine (GC) content (A) and simple sequence repeats (SSRs) (B) identified in transcriptome of** ***S. hexandrum*** **.** (DOC 60 KB)

Additional file 8: **Gene Ontology (GO) classification for** ***S. hexandrum*** **transcripts in cellular component, molecular function and biological process categories.** (TIFF 3 MB)

Additional file 9: **Functional characterization and abundance of** ***S. hexandrum*** **transcriptome for enzyme classes (A), and Kyoto Encyclopedia of Genes and Genomes (KEGG) pathways (B).** *S. hexandrum* transcripts were classified in top 50 abundant enzyme classes and KEGG pathways. (TIFF 5 MB)

Additional file 10: **Number of contigs in different Functional Catalogue (FunCat) categories at 15°C and 25°C.** (XLS 22 KB)

Additional file 11: **Transcription factor (TF) families identified in** ***S. hexandrum*** **transcriptome.** (TIFF 397 KB)

Additional file 12: **Fragments per kilobase of exon per million fragments mapped (FPKM) based expression of** ***transcription factors (TFs)*** **identified in** ***S. hexandrum*** **transcriptome at 15°C and 25°C.** (XLS 18 KB)

Additional file 13: **Fragments per kilobase of exon per million fragments mapped (FPKM) based expression analysis of** ***cytochrome P450s*** **(** ***CYPs*** **, Sheet 1),** ***methyltransferases*** **(** ***MTs*** **, Sheet 2) and** ***uridine diphosphate glycosyltransferases*** **(** ***UGTs*** **, Sheet 3) in** ***S. hexandrum*** **transcriptome.** (XLS 52 KB)

Additional file 14: **Fragments per kilobase of exon per million fragments mapped (FPKM) based expression analysis of** ***cytochrome P450s*** **(** ***CYPs*** **) in** ***S. hexandrum*** **transcriptome.** Expression of 39 CYPs was studied at 15°C and 25°C. Details of the corresponding contigs listed in Additional file 13. (TIF 3 MB)

Additional file 15: **Fragments per kilobase of exon per million fragments mapped (FPKM) based expression analysis of** ***methyltransferases*** **(** ***MTs*** **) in** ***S. hexandrum*** **transcriptome.** Expression of 18 MTs was studied at 15°C and 25°C. Details of the corresponding contigs are listed in Additional file 13. (TIF 1 MB)

Additional file 16: **Fragments per kilobase of exon per million fragments mapped (FPKM) based expression analysis of** ***uridine diphosphate glycosyltransferases*** **(** ***UGTs*** **) in** ***S. hexandrum*** **transcriptome.** Expression of 10 UGTs was studied at 15°C and 25°C. Details of the corresponding contigs are listed in Additional file 13. (TIF 2 MB)

## References

[1] Shaw JMH (2009). New combinations for the varieties of *Sinopodophyllum hexandrum*. Hanburyana.

[2] Stähelin HF, von Wartburg A (1991). The chemical and biological route from podophyllotoxin glucoside to etoposide: ninth Cain memorial Award lecture. Cancer Res.

[3] Nadeem M, Palni LMS, Purohit AN, Pandey H, Nandi SK (2000). Propagation and conservation of *Podophyllum hexandrum* Royle: an important medicinal herb. Biol Conserv.

[4] Giri A, Lakshmi Narasu M (2000). Production of podophyllotoxin from *Podophyllum hexandrum*: a potential natural product for clinically useful anticancer drugs. Cytotechnology.

[5] You Y (2005). Podophyllotoxin derivatives: current synthetic approaches for new anticancer agents. Curr Pharm Des.

[6] Kim E, Donohue K (2013). Local adaptation and plasticity of *Erysimum capitatum* to altitude: its implications for responses to climate change. J Ecol.

[7] Kushwaha R, Pandey S, Chanda S, Bhattacharya A, Ahuja PS (2008). Temperature dependent growth and emergence of functional leaves: an adaptive mechanism in the seedlings of the western Himalayan plant *Podophyllum hexandrum*. J Plant Res.

[8] Singh A, Purohit AN (1997). Light and temperature effects on physiological reactions on alpine and temperate populations of *Podophyllum hexandrum* Royle. J Herbs Spices Med Plants.

[9] Schrader J, Moyle R, Bhalerao R, Hertzberg M, Lundeberg J, Nilsson P, Bhalerao RP (2004). Cambial meristem dormancy in trees involves extensive remodelling of the transcriptome. Plant J.

[10] Derory J, Léger P, Garcia V, Schaeffer J, Hauser MT, Salin F, Luschnig C, Plomion C, Glössl J, Kremer A (2006). Transcriptome analysis of bud burst in sessile oak (*Quercus petraea*). New Phytol.

[11] Paul A, Kumar S (2011). Responses to winter dormancy, temperature, and plant hormones share gene networks. Funct Integr Genomics.

[12] Gahlan P, Singh HR, Shankar R, Sharma N, Kumari A, Chawla V, Ahuja PS, Kumar S (2012). *De novo* sequencing and characterization of *Picrorhiza kurrooa* transcriptome at two temperatures showed major transcriptome adjustments. BMC Genomics.

[13] Venkataraman K (2009). India’s Biodiversity Act 2002 and its role in conservation. Trop Ecol.

[14] Bhattacharyya D, Sinha R, Ghanta S, Chakraborty A, Hazra S, Chattopadhyay S (2012). Proteins differentially expressed in elicited cell suspension culture of *Podophyllum hexandrum* with enhanced podophyllotoxin content. Proteome Sci.

[15] Sultan P, Shawl AS, Abdellah AA, Ramteke PW (2010). Isolation, Characterization and Comparative Study on Podophyllotoxin and Related Glycosides of *Podophyllum heaxandrum*. Curr Res J Biol Sci.

[16] Marques JV, Kim KW, Lee C, Costa MA, May GD, Crow JA, Davin LB, Lewis NG (2013). Next generation sequencing in predicting gene function in podophyllotoxin biosynthesis. J Biol Chem.

[17] **Compound extraction of*****Podophyllum peltatum*** [http://uic.edu/pharmacy/MedPlTranscriptome/d_podo_p_3.html]

[18] Muoki RC, Paul A, Kumari A, Singh K, Kumar S (2012). An improved protocol for the isolation of RNA from roots of tea (*Camellia sinensis* (L.) O. Kuntze). Mol Biotechnol.

[19] **GenScript real-time PCR primer design** [https://www.genscript.com/ssl-bin/app/primer]

[20] Pfaffl WP, Horgan GW, Dempfle L (2002). Relative expression software tool (REST©) for group-wise comparison and statistical analysis of relative expression results in real-time PCR. Nucleic Acids Res.

[21] Li R, Zhu H, Ruan J, Qian W, Fang X, Shi Z, Li Y, Li S, Shan G, Kristiansen K, Li S, Yang H, Wang J, Wang J (2009). *De nov*o assembly of human genomes with massively parallel short read sequencing. Genome Res.

[22] Li W, Godzik A (2006). Cd-hit: a fast program for clustering and comparing large sets of protein or nucleotide sequences. Bioinformatics.

[23] UniProt Consortium (2011). Reorganizing the protein space at the Universal Protein Resource (UniProt). Nucleic Acids Res.

[24] Ashburner M, Ball CA, Blake JA, Botstein D, Butler H, Cherry JM, Davis AP, Dolinski K, Dwight SS, Eppig JT, Harris MA, Hill DP, Issel-Tarver L, Kasarskis A, Lewis S, Matese JC, Richardson JE, Ringwald M, Rubin GM, Sherlock G (2000). Gene ontology: tool for the unification of biology. The Gene Ontology Consortium. Nat Genet.

[25] Ogata H, Goto S, Sato K, Fujibuchi W, Bono H, Kanehisa M (1999). KEGG: Kyoto Encyclopedia of Genes and Genomes. Nucleic Acids Res.

[26] Webb EC (1992). International Union of Biochemistry and Molecular Biology. Nomenclature Committee. Enzyme nomenclature: Recommendations of the Nomenclature Committee of the International Union of Biochemistry and Molecular Biology on the nomenclature and classification of enzymes.

[27] Schmid R, Blaxter ML (2008). annot8r: GO, EC and KEGG annotation of EST datasets. BMC Bioinformatics.

[28] Pérez-Rodríguez P, Riaño-Pachón DM, Corrêa LG, Rensing SA, Kersten B, Mueller-Roeber B (2010). PlnTFDB: updated content and new features of the plant transcription factor database. Nucleic Acids Res.

[29] Ruepp A, Zollner A, Maier D, Albermann K, Hani J, Mokrejs M, Tetko I, Güldener U, Mannhaupt G, Münsterkötter M, Mewes HW (2004). The FunCat, a functional annotation scheme for systematic classification of proteins from whole genomes. Nucleic Acids Res.

[30] Lamesch P, Berardini TZ, Li D, Swarbreck D, Wilks C, Sasidharan R, Muller R, Dreher K, Alexander DL, Garcia-Hernandez M, Karthikeyan AS, Lee CH, Nelson WD, Ploetz L, Singh S, Wensel A, Huala E (2012). The Arabidopsis Information Resource (TAIR): improved gene annotation and new tools. Nucleic Acids Res.

[31] Marchler-Bauer A, Lu S, Anderson JB, Chitsaz F, Derbyshire MK, DeWeese-Scott C, Fong JH, Geer LY, Geer RC, Gonzales NR, Gwadz M, Hurwitz DI, Jackson JD, Ke Z, Lanczycki CJ, Lu F, Marchler GH, Mullokandov M, Omelchenko MV, Robertson CL, Song JS, Thanki N, Yamashita RA, Zhang D, Zhang N, Zheng C, Bryant SH (2011). CDD: a Conserved domain database for the functional annotation of proteins. Nucleic Acids Res.

[32] Trapnell C, Pachter L, Salzberg SL (2009). TopHat: discovering splice junctions with RNA-seq. Bioinformatics.

[33] Trapnell C, Williams BA, Pertea G, Mortazavi A, Kwan G, van Baren MJ, Salzberg SL, Wold BJ, Pachter L (2010). Transcript assembly and quantification by RNA-Seq reveals unannotated transcripts and isoform switching during cell differentiation. Nat Biotechnol.

[34] **MISA Program** [http://pgrc.ipk-gatersleben.de/misa]

[35] Zhang P, Dreher K, Karthikeyan A, Chi A, Pujar A, Caspi R, Karp P, Kirkup V, Latendresse M, Lee C, Mueller LA, Muller R, Rhee SY (2010). Creation of a genome-wide metabolic pathway database for *Populus trichocarpa* using a new approach for reconstruction and curation of metabolic pathways for plants. Plant Physiol.

[36] Salick J, Fangb Z, Byg A (2009). Eastern Himalayan alpine plant ecology, Tibetan ethnobotany, and climate change. Global Environ Chang.

[37] Zobayed SM, Afreen F, Kozai T (2005). Temperature stress can alter the photosynthetic efficiency and secondary metabolite concentrations in St. John’s wort. Plant Physiol Biochem.

[38] Alam MA, Gulati P, Aswini KG, Gyan PM, Pradeep KN (2009). Assessment of genetic diversity among *Podophyllum hexandrum* genotypes of northwestern Himalayan region for podophyllotoxin production. Indian J Biotechnol.

[39] Chandra S (2004). Effect of altitude on energy exchange characteristics of some alpine medicinal crops from central Himalayas. J Agron Crop Sci.

[40] Altschul SF, Gish W, Miller W, Myers EW, Lipman DJ (1990). Basic local alignment search tool. J Mol Biol.

[41] Vinogradov AE (2003). DNA helix: the importance of being GC-rich. Nucleic Acids Res.

[42] Nestor CE, Monckton DG (2011). Correlation of inter-locus polyglutamine toxicity with CAG•CTG triplet repeat expandability and flanking genomic DNA GC content. PLoS One.

[43] Holland JB, Helland SJ, Sharopova N, Rhyne DC (2001). Polymorphism of PCR-based markers targeting exons, introns, promoter regions, and SSRs in maize and introns and repeat sequences in oat. Genome.

[44] Musashi M, Ota S, Shiroshita N (2000). The role of protein kinase C isoforms in cell proliferation and apoptosis. Int J Hematol.

[45] Annadurai RS, Jayakumar V, Mugasimangalam RC, Katta MA, Anand S, Gopinathan S, Sarma SP, Fernandes SJ, Mullapudi N, Murugesan S, Rao SN (2012). Next generation sequencing and *de novo* transcriptome analysis of *Costus pictus* D. Don, a non-model plant with potent anti-diabetic properties. BMC Genomics.

[46] Smith IK, Polle A, Rennenberg H, Alscher RG, Cumming JR (1992). Glutathione. Stress Responses in Plants: Adaptation and Acclimation Mechanisms.

[47] Vu JCV, Allen LH, Gesch RW (2006). Up-regulation of photosynthesis and sucrose metabolism enzymes in young expanding leaves of sugarcane under elevated growth CO_2_. Plant Sci.

[48] An D, Yang J, Zhang P (2012). Transcriptome profiling of low temperature treated cassava apical shoots showed dynamic responses of tropical plant to cold stress. BMC Genomics.

[49] Hotton SK, Callis J (2008). Regulation of cullin RING ligases. Annu Rev Plant Biol.

[50] **TAIR** [https://www.arabidopsis.org/servlets/TairObject?name=AT3G12670&type=locus]

[51] Henriksson E, Olsson AS, Johannesson H, Johansson H, Hanson J, Engström P, Söderman E (2005). Homeodomain leucine zipper class I genes in Arabidopsis. Expression patterns and phylogenetic relationships. Plant Physiol.

[52] Ha CM, Jun JH, Nam HG, Fletcher JC (2004). BLADE-ON-PETIOLE1 encodes a BTB/POZ domain protein required for leaf morphogenesis in *Arabidopsis thaliana*. Plant Cell Physiol.

[53] Jofuku KD, den Boer BG, Van Montagu M, Okamuro JK (1994). Control of Arabidopsis flower and seed development by the homeotic gene *APETALA*2. Plant Cell.

[54] Samach A, Onouchi H, Gold SE, Ditta GS, Schwarz-Sommer Z, Yanofsky MF, Coupland G (2000). Distinct roles of CONSTANS target genes in reproductive development of *Arabidopsis*. Science.

[55] Kim WC, Reca IB, Kim Y, Park S, Thomashow MF, Keegstra K, Han KH (2014). Transcription factors that directly regulate the expression of CSLA9 encoding mannan synthase in *Arabidopsis thaliana*. Plant Mol Biol.

[56] Apuya NR, Yadegari R, Fischer RL, Harada JJ, Zimmerman JL, Goldberg RB (2001). The Arabidopsis embryo mutant schlepperless has a defect in the *chaperonin-60alpha* gene. Plant Physiol.

[57] Davletova S, Schlauch K, Coutu J, Mittler R (2005). The zinc-finger protein Zat12 plays a central role in reactive oxygen and abiotic stress signaling in *Arabidopsis*. Plant Physiol.

[58] Polidoros AN, Mylona PV, Pasentsis K, Scandalios JG, Tsaftaris AS (2005). The maize alternative oxidase 1a (*Aox1a*) gene is regulated by signals related to oxidative stress. Redox Rep.

[59] Diédhiou CJ, Popova OV, Dietz KJ, Golldack D (2008). The SNF1-type serine-threonine protein kinase SAPK4 regulates stress-responsive gene expression in rice. BMC Plant Biol.

[60] Wang W, Vinocur B, Shoseyov O, Altman A (2004). Role of plant heat-shock proteins and molecular chaperones in the abiotic stress response. Trends Plant Sci.

[61] Mizoi J, Shinozaki K, Yamaguchi-Shinozaki K (2012). AP2/ERF family transcription factors in plant abiotic stress responses. Biochim Biophys Acta.

[62] Yi Y, Guerinot ML (1996). Genetic evidence that induction of root Fe(III) chelate reductase activity is necessary for iron uptake under iron deficiency. Plant J.

[63] Sun S, Yu JP, Chen F, Zhao TJ, Fang XH, Li YQ, Sui SF (2008). TINY, a dehydration-responsive element (DRE)-binding protein-like transcription factor connecting the DRE- and ethylene-responsive element-mediated signaling pathways in *Arabidopsis*. J Biol Chem.

[64] Wang J, Chen L, Huang S, Liu J, Ren X, Tian X, Qiao J, Zhang W (2012). RNA-seq based identification and mutant validation of gene targets related to ethanol resistance in cyanobacterial *Synechocystis* sp. PCC 6803. Biotechnol Biofuels.

[65] Xu L, Zhu L, Tu L, Liu L, Yuan D, Jin L, Long L, Zhang X (2011). Lignin metabolism has a central role in the resistance of cotton to the wilt fungus *Verticillium dahliae* as revealed by RNA-Seq-dependent transcriptional analysis and histochemistry. J Exp Bot.

[66] Heim MA, Jakoby M, Werber M, Martin C, Weisshaar B, Bailey PC (2003). The basic helix-loop-helix transcription factor family in plants: a genome-wide study of protein structure and functional diversity. Mol Biol Evol.

[67] Dubos C, Stracke R, Grotewold E, Weisshaar B, Martin C, Lepiniec L (2010). MYB transcription factors in *Arabidopsis*. Trends Plant Sci.

[68] Bienz M (2006). The PHD finger, a nuclear protein-interaction domain. Trends Biochem Sci.

[69] Tran LS, Nakashima K, Sakuma Y, Simpson SD, Fujita Y, Maruyama K, Fujita M, Seki M, Shinozaki K, Yamaguchi-Shinozaki K (2004). Isolation and functional analysis of *Arabidopsis* stress-inducible NAC transcription factors that bind to a drought-responsive cis-element in the early responsive to dehydration stress 1 promoter. Plant Cell.

[70] Englbrecht CC, Schoof H, Böhm S (2004). Conservation, diversification and expansion of C2H2 zinc finger proteins in the *Arabidopsis thaliana* genome. BMC Genomics.

[71] Jackson DE, Dewick PM (1984). Biosynthesis of Podophyllum lignans-II. Interconversion of aryltetralin lignans in *Podophyllum hexandrum*. Phytochemistry.

[72] Hoffmann L, Maury S, Martz F, Geoffroy P, Legrand M (2003). Purification, cloning and properties of an acyltransferase controlling shikimate and quinate ester intermediates in phenylpropanoid metabolism. J Biol Chem.

[73] Schoch G, Goepfert S, Morant M, Hehn A, Meyer D, Ullmann P, Werck-Reichhart D (2001). CYP98A3 from *Arabidopsis thaliana* is a 3′-hydroxylase of phenolic esters, a missing link in the phenylpropanoid pathway. J Biol Chem.

[74] Fraser CM, Chapple C (2011). The phenylpropanoid pathway in *Arabidopsis*. Arabidopsis Book.

[75] Davin LB, Wang HB, Crowell AL, Bedgar DL, Martin DM, Sarkanen S, Lewis NG (1997). Stereoselective bimolecular phenoxy radical coupling by an auxiliary (dirigent) protein without an active center. Science.

[76] Katayama T, Davin LB, Lewis NG (1992). An extraordinary accumulation of (-)-pinoresinol in cell-free extracts of *Forsythia intermedia*: evidence for enantiospecific reduction of (+)-pinoresinol. Phytochemistry.

[77] Katayama T, Davin LB, Chu A, Lewis NG (1993). Novel benzylic ether reductions in lignan biogenesis in *Forsythia intermedia*. Phytochemistry.

[78] Umezawa T, Davin LB, Lewis NG (1991). Formation of lignans, (-)-secoisolariciresinol and (-)-matairesinol with *Forsythia intermedia* cell-free extracts. J Biol Chem.

[79] Sakakibara N, Suzuki S, Umezawa T, Shimada M (2003). Biosynthesis of yatein in *Anthriscus sylvestris*. Org Biomol Chem.

[80] Broomhead AJ, Rahman MA, Dewick PM, Jackson DE, Lucas JA (1991). Matairesinol as precursor of *Podophyllum lignans*. Phytochemistry.

[81] Kuhlmann S, Kranz K, Lücking B (2002). Aspects of cytotoxic lignan biosynthesis in suspension cultures of *Linum nodiflorum*. Phytochem Rev.

[82] Kamil WM, Dewick PM (1986). Biosynthesis of the lignans α- and β-peltatin. Phytochemistry.

[83] van Uden W, Bouma AS, Bracht Waker JF, Middel O, Wichers HJ, Waard PD, Woerdenbag HJ, Kellogg RM, Pras N (1995). The production of podophyllotoxin and its 5-methoxy derivative through bioconversion of cyclodextrin complexed deoxy-podophyllotoxin by plant cell cultures. Plant Cell Tissue Organ Cult.

[84] van Uden W, Bos JA, Boeke GM, Woerdenbag HJ, Pras N (1997). The large-scale isolation of deoxypodophyllotoxin from rhizomes of *Anthriscus sylvestris* followed by its bioconversion into 5-methoxypodophyllotoxin β-d-glucoside by cell cultures of *Linun flavum*. J Natural Prod.

[85] Yousefzadi M, Sharifi M, Behmanesh M, Moyano E, Bonfill M, Cusido RM, Palazon J (2010). Podophyllotoxin: current approaches to its biotechnological production and future challenges. Eng Life Sci.

[86] Singh K, Rani A, Paul A, Dutt S, Joshi R, Gulati A, Ahuja PS, Kumar S (2009). Differential display mediated cloning of *anthocyanidin reductase* gene from tea (*Camellia sinensis*) and its relationship with the concentration of epicatechins. Tree Physiol.

[87] Kawoosa T, Singh H, Kumar A, Sharma SK, Devi K, Dutt S, Vats SK, Sharma M, Ahuja PS, Kumar S (2010). Light and temperature regulated terpene biosynthesis: hepatoprotective monoterpene picroside accumulation in *Picrorhiza kurrooa*. Funct Integr Genomics.

[88] Kumar H, Singh K, Kumar S (2012). 2C-methyl-d-erythritol-2,4-cyclodiphosphate synthase from *Stevia rebaudiana* Bertoni is a functional gene. Mol Biol Rep.

[89] Singh RS, Gara RK, Bhardwaj PK, Kaachra A, Malik S, Kumar R, Sharma M, Ahuja PS, Kumar S (2010). Expression of 3-hydroxy-3-methylglutaryl-CoA reductase, p-hydroxybenzoate-m-geranyltransferase and genes of phenylpropanoid pathway exhibits positive correlation with shikonins content in arnebia [*Arnebia euchroma* (Royle) Johnston]. BMC Mol Biol.

[90] Schuler MA (1996). Plant cytochrome P450 monooxygenases. Crit Rev Plant Sci.

[91] Federolf K, Alfermann AW, Fuss E (2007). Aryltetralin-lignan formation in two different cell suspension cultures of *Linum album*: Deoxypodophyllotoxin 6-hydroxylase, a key-enzyme in the formation of 6-methoxypodophyllotoxin. Phytochemistry.

[92] Lam KC, Ibrahim RK, Behdad B, Dayanandan S (2007). Structure, function, and evolution of plant O-methyltransferases. Genome.

[93] Lewis NG, Yamamoto E (1990). Lignin: occurrence, biogenesis and biodegradation. Annu Rev Plant Physiol Plant Mol Biol.

[94] Boerjan W, Ralph J, Baucher M (2003). Lignin biosynthesis. Annu Rev Plant Biol.

[95] Zhong R, Morrison WH, Himmelsbach DS, Poole FL, Ye ZH (2000). Essential role of caffeoyl coenzyme A O-methyltransferase in lignin biosynthesis in woody poplar plants. Plant Physiol.

[96] Ross J, Li Y, Lim E, Bowles DJ (2001). Higher plant glycosyltransferases. Genome Biol.

[97] Vogt T, Jones P (2000). Glycosyltransferases in plant natural product synthesis: characterization of a supergene family. Trends Plant Sci.

[98] Wang J, Hou B (2009). Glycosyltransferases: key players involved in the modification of plant secondary metabolites. Front Biol China.

[99] Berim A, Ebel R, Schneider B, Petersen M (2008). UDP-glucose:(6-methoxy) podophyllotoxin 7-O-glucosyltransferase from suspension cultures of *Linum nodiflorum*. Phytochemistry.

[100] Jha A, Mehra M, Shankar R (2011). The regulatory epicenter of miRNAs. J Biosci.

[101] Jha A, Shankar R (2013). miReader: Discovering novel miRNAs in species without sequenced genome. PLoS One.

