# Supplementary figures and images for: Transcriptome sequencing of rhizome tissue of Sinopodophyllum hexandrum at two temperatures
Source: BMC Genomics. 2014 Oct 7;15(1):871. doi: 10.1186/1471-2164-15-871 (PMC4200142; doi:10.1186/1471-2164-15-871)

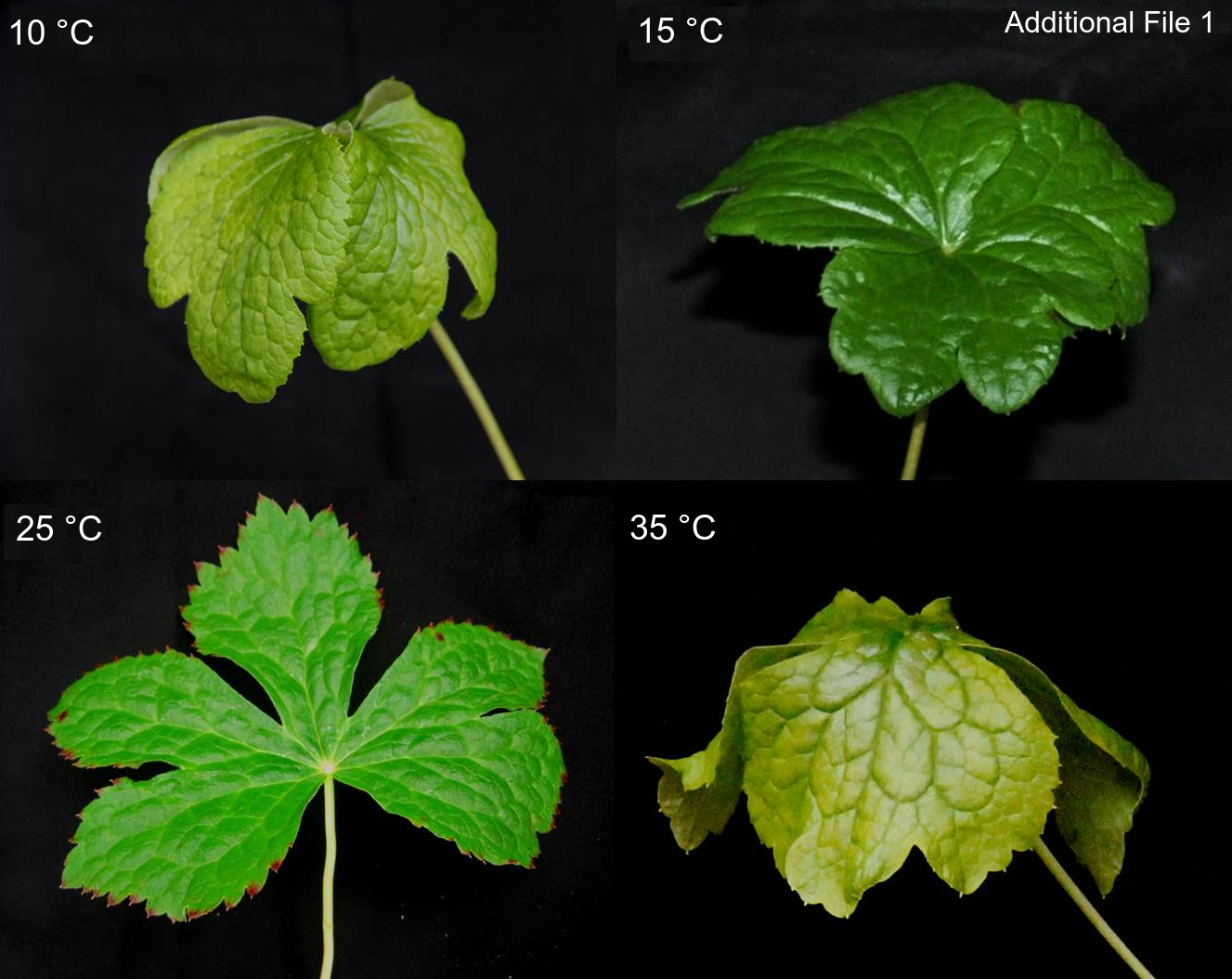

Supplement: Supplementary file 1 — Additional file 1: Leaf morphology of S. hexandrum grown at 10°C, 15°C, 25°C and 35°C for 21 days. (PNG 1 MB) [file 12864_2013_6550_MOESM1_ESM.png]

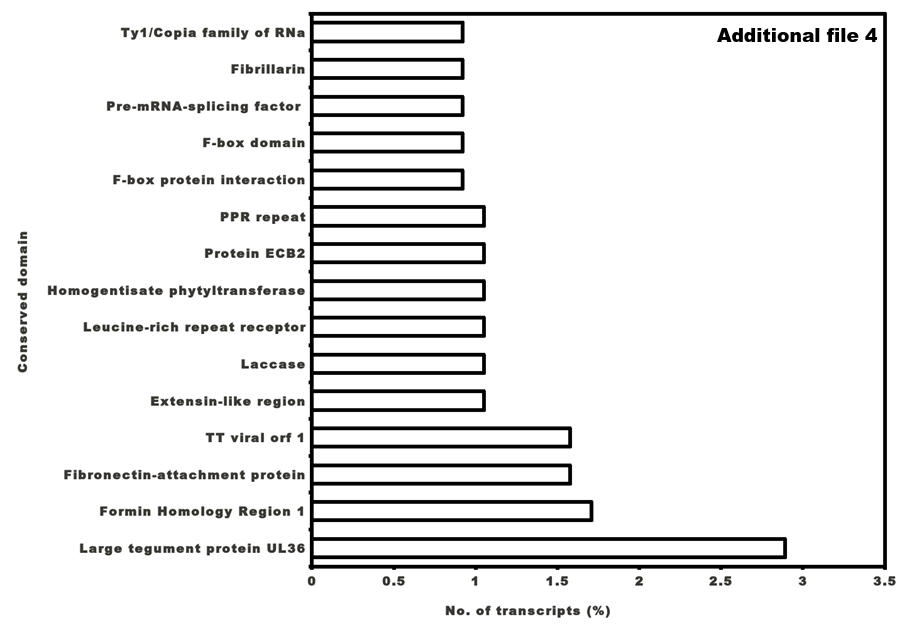

Supplement: Supplementary file 4 — Additional file 4: Top 15 conserved domains identified by RPS-BLAST in S. hexandrum transcriptome. (TIFF 2 MB) [file 12864_2013_6550_MOESM4_ESM.tiff]

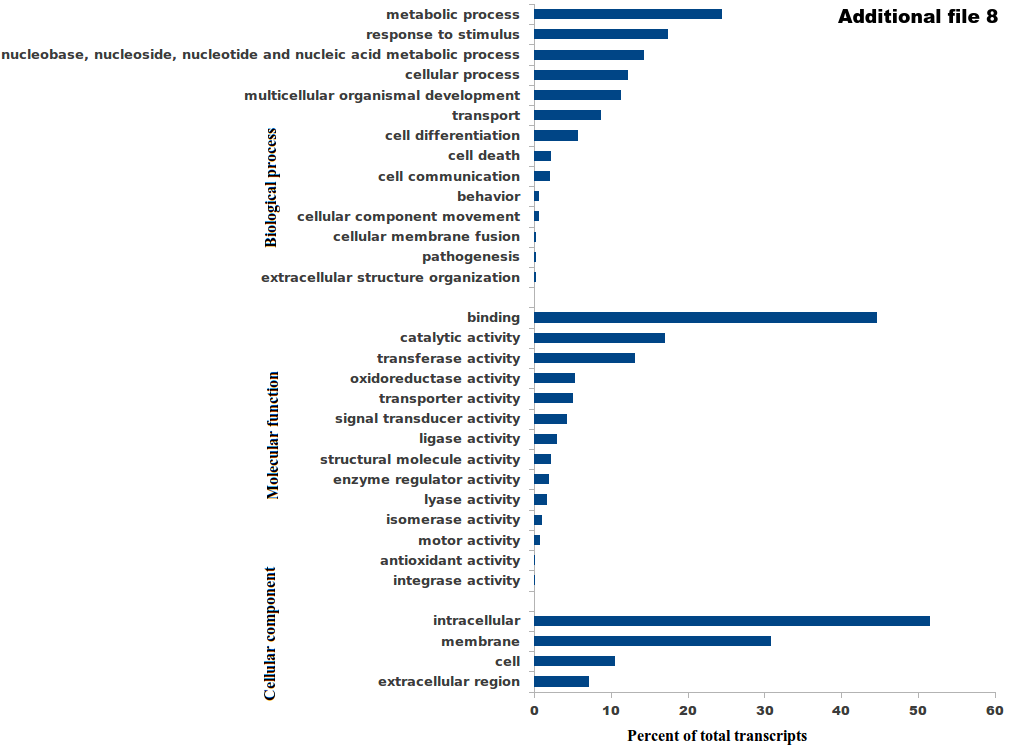

Supplement: Supplementary file 8 — Additional file 8: Gene Ontology (GO) classification for S. hexandrum transcripts in cellular component, molecular function and biological process categories. (TIFF 3 MB) [file 12864_2013_6550_MOESM8_ESM.tiff]

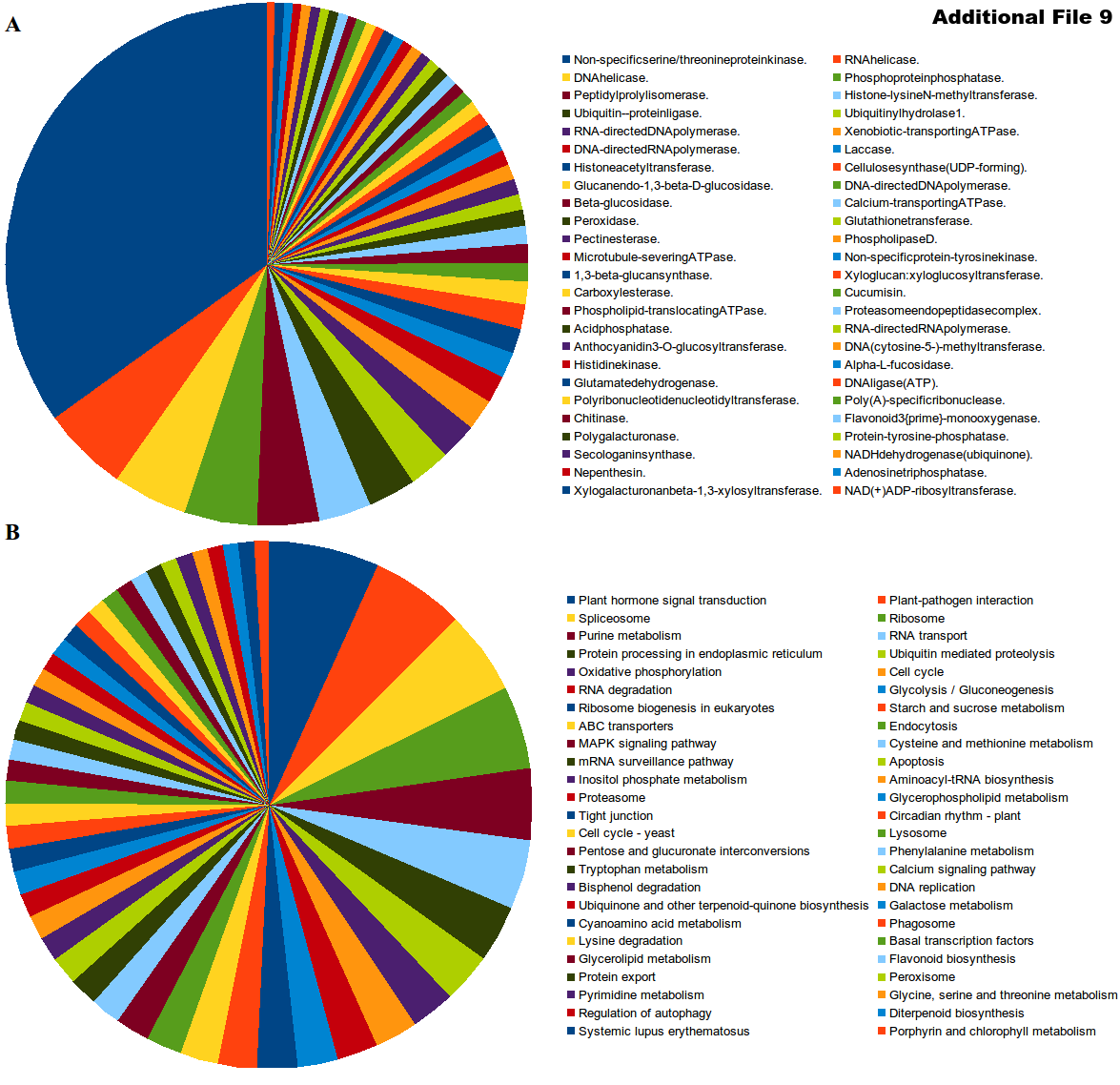

Supplement: Supplementary file 9 — Additional file 9: Functional characterization and abundance of S. hexandrum transcriptome for enzyme classes (A), and Kyoto Encyclopedia of Genes and Genomes (KEGG) pathways (B). S. hexandrum transcripts were classified in top 50 abundant enzyme classes and KEGG pathways. (TIFF 5 MB) [file 12864_2013_6550_MOESM9_ESM.tiff]

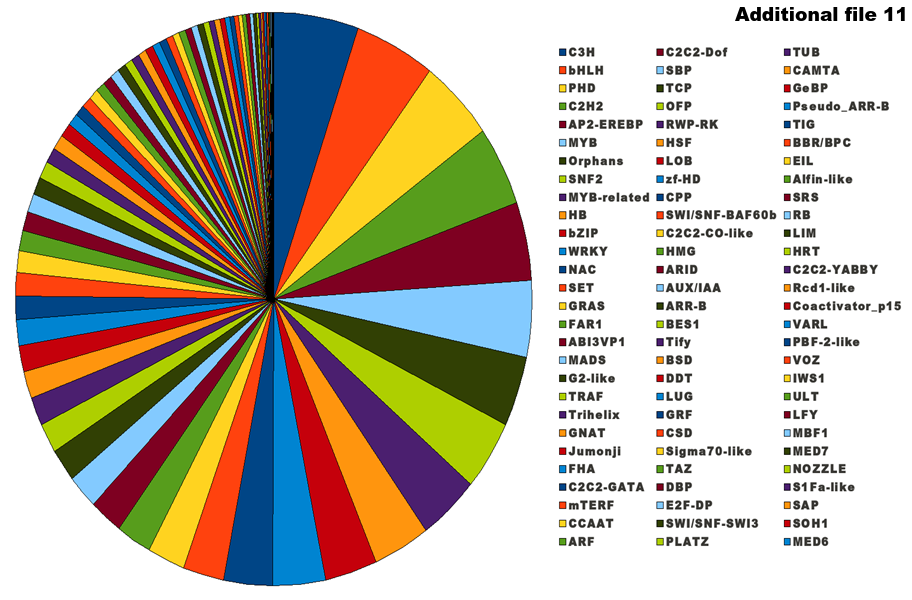

Supplement: Supplementary file 11 — Additional file 11: Transcription factor (TF) families identified in S. hexandrum transcriptome. (TIFF 397 KB) [file 12864_2013_6550_MOESM11_ESM.tiff]

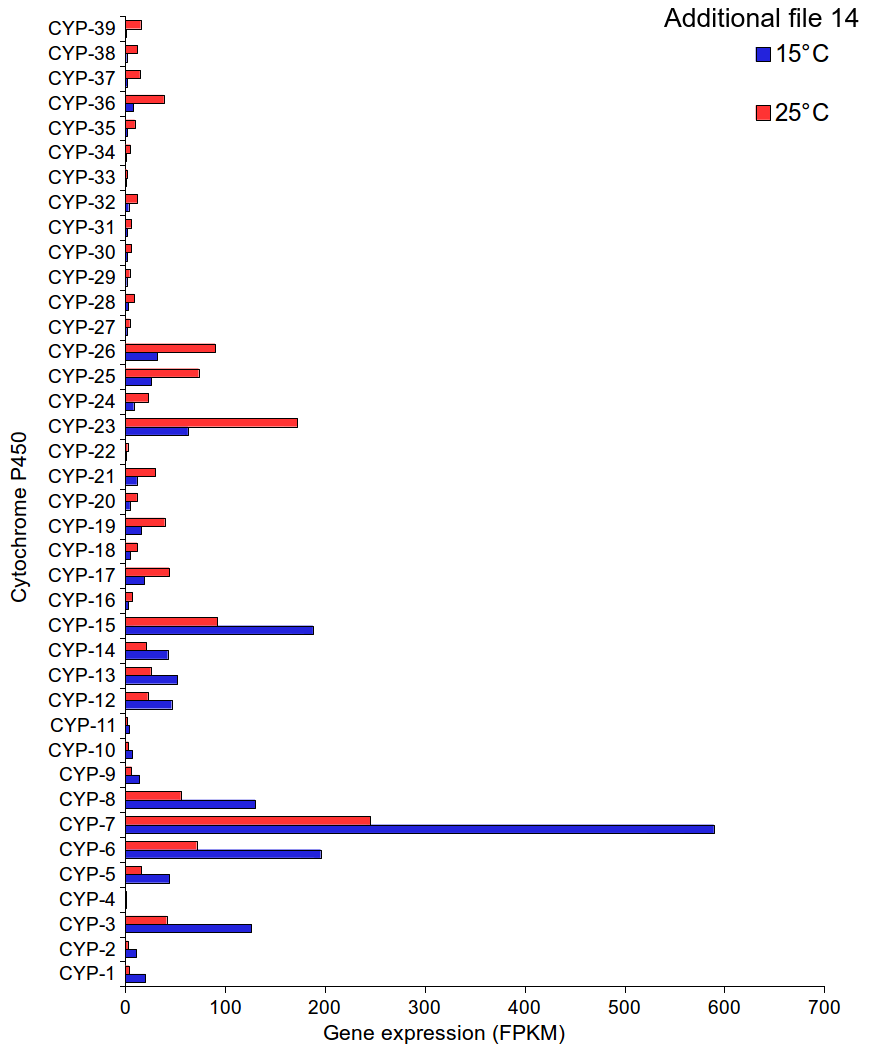

Supplement: Supplementary file 14 — Additional file 14: Fragments per kilobase of exon per million fragments mapped (FPKM) based expression analysis of cytochrome P450s ( CYPs ) in S. hexandrum transcriptome. Expression of 39 CYPs was studied at 15°C and 25°C. Details of the corresponding contigs listed in Additional file 13. (TIF 3 MB) [file 12864_2013_6550_MOESM14_ESM.tif]

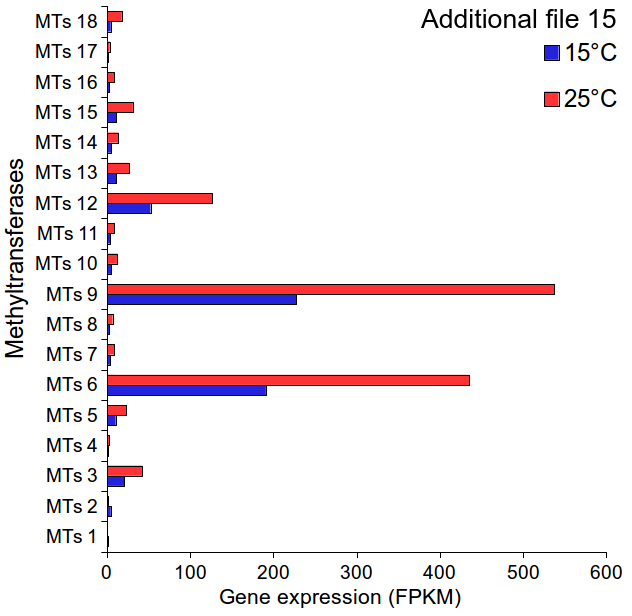

Supplement: Supplementary file 15 — Additional file 15: Fragments per kilobase of exon per million fragments mapped (FPKM) based expression analysis of methyltransferases ( MTs ) in S. hexandrum transcriptome. Expression of 18 MTs was studied at 15°C and 25°C. Details of the corresponding contigs are listed in Additional file 13. (TIF 1 MB) [file 12864_2013_6550_MOESM15_ESM.tif]

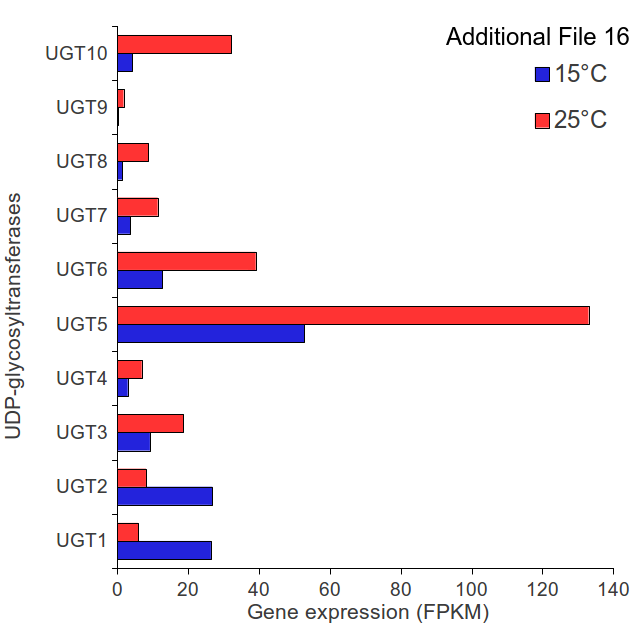

Supplement: Supplementary file 16 — Additional file 16: Fragments per kilobase of exon per million fragments mapped (FPKM) based expression analysis of uridine diphosphate glycosyltransferases ( UGTs ) in S. hexandrum transcriptome. Expression of 10 UGTs was studied at 15°C and 25°C. Details of the corresponding contigs are listed in Additional file 13. (TIF 2 MB) [file 12864_2013_6550_MOESM16_ESM.tif]
