# Supplementary material for: Transcriptome sequencing of rhizome tissue of Sinopodophyllum hexandrum at two temperatures
Source: BMC Genomics. 2014 Oct 7;15(1):871. doi: 10.1186/1471-2164-15-871 (PMC4200142; doi:10.1186/1471-2164-15-871)
Supplement: Supplementary file 3 — Additional file 3: Effect of k-mer size on assembling performance of transcriptome. (DOC 31 KB) [file 12864_2013_6550_MOESM3_ESM.doc]

**Additional file 3: Effect of k-mer size on assembling performance of transcriptome.**

| K- mer | Average Coverage | Average Length (bp) | Max. Length  (bp) | Total Transcripts | % of transcripts above 1000 bp |
| --- | --- | --- | --- | --- | --- |
| 19 | 85.03 | 537.26 | 11,422 | 69,788 | 14.77 |
| 21 | 86.13 | 543.63 | 10,869 | 65,228 | 15.22 |
| 23 | 88.34 | 543.11 | 14,390 | 60,089 | 15.57 |
| 25 | 91.69 | 533.84 | 11,441 | 54,276 | 15.37 |
| 27 | 95.36 | 506.61 | 7,871 | 47,043 | 14.35 |
| 29 | 107.9 | 449.98 | 7,488 | 36,094 | 11.53 |
