# Supplementary material for: Transcriptome sequencing of rhizome tissue of Sinopodophyllum hexandrum at two temperatures
Source: BMC Genomics. 2014 Oct 7;15(1):871. doi: 10.1186/1471-2164-15-871 (PMC4200142; doi:10.1186/1471-2164-15-871)

**Additional File 7A: GC content of *S. hexandrum* transcriptome**

| **GC content range** | **Number of transcripts** | **Percent of transcripts** |
| --- | --- | --- |
| >80% | 0 | 0 |
| 70-79% | 1 | 0.0019599773 |
| 60-69% | 78 | 0.1528782266 |
| 50-59% | 3697 | 7.246035946 |
| 40-49% | 42851 | 83.986985751 |
| 30-39% | 4371 | 8.5670606221 |
| 20-29% | 23 | 0.0450794771 |
| 10-19% | 0 | 0 |
| 0-9% | 0 | 0 |


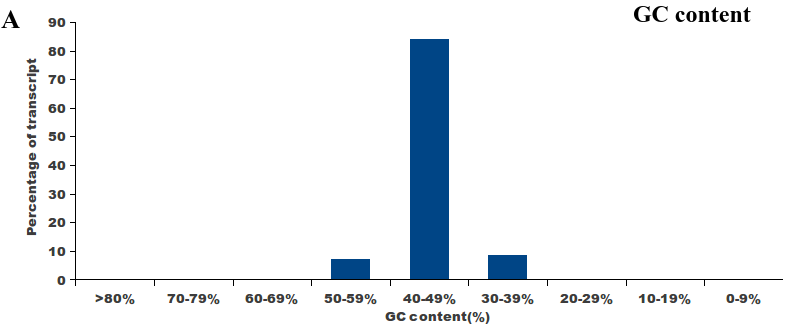


**Additional File 7B. Short sequence repeats (SSRs) identified in transcripts of *S. hexandrum***

| **SSR mining** | |
| --- | --- |
| Total number of sequences examined: | 59,244 |
| Total size of examined sequences (bp): | 3,24,71,839 |
| Total number of identified SSRs: | 3,226 |
| Number of SSR containing sequences: | 2,897 (4.89%) |
| Number of sequences containing more than 1 SSR: | 286 |
| Number of SSRs present in compound formation: | 111 |
| **Distribution of SSRs in different repeat types** | |
| **Unit size** | **Number of SSRs** |
| Mono-nucleotide | 561 (19.36%) |
| Di-nucleotide | 1054 (36.38%) |
| Tri-nucleotide | 1576 (54.40%) |
| Tetra-nucleotide | 32 (1.10%) |
| Penta-nucleotide | Nill |
| Hexa-nucleotide | 3 (0.10%) |


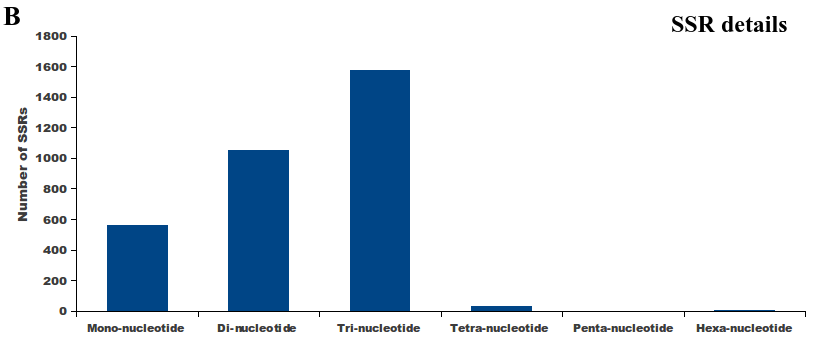

Supplement: Supplementary file 7 — Additional file 7: Guanine-cytosine (GC) content (A) and simple sequence repeats (SSRs) (B) identified in transcriptome of S. hexandrum . (DOC 60 KB) [file 12864_2013_6550_MOESM7_ESM.doc]
